# Free-energy minimization in joint agent-environment systems: A niche construction perspective

**DOI:** 10.1016/j.jtbi.2018.07.002

**Published:** 2018-10-14

**Authors:** Jelle Bruineberg, Erik Rietveld, Thomas Parr, Leendert van Maanen, Karl J Friston

**Affiliations:** a Department of Philosophy, Institute for Logic, Language and Computation, University of Amsterdam, The Netherlands; b Amsterdam Brain and Cognition Centre, University of Amsterdam, The Netherlands; c Wellcome Trust Centre for Neuroimaging, Institute of Neurology, University College London, London WC1N 3BG, UK; d Academic Medical Center, Department of Psychiatry, University of Amsterdam, The Netherlands; e Department of Psychology, University of Amsterdam, The Netherlands; f Department of Philosophy, University of Twente, The Netherlands

**Keywords:** Active inference, Free energy principle, Markov decision processes, Niche construction, Agent-environment complementarity, Adaptive environments, Desire paths

## Abstract

The free-energy principle is an attempt to explain the structure of the agent and its brain, starting from the fact that an agent exists (Friston and Stephan, 2007; Friston et al., 2010). More specifically, it can be regarded as a systematic attempt to understand the ‘fit’ between an embodied agent and its niche, where the quantity of free-energy is a measure for the ‘misfit’ or disattunement (Bruineberg and Rietveld, 2014) between agent and environment. This paper offers a proof-of-principle simulation of niche construction under the free-energy principle. Agent-centered treatments have so far failed to address situations where environments change alongside agents, often due to the action of agents themselves. The key point of this paper is that the minimum of free-energy is not at a point in which the agent is maximally adapted to the statistics of a static environment, but can better be conceptualized an attracting manifold within the joint agent-environment state-space as a whole, which the system tends toward through mutual interaction. We will provide a general introduction to active inference and the free-energy principle. Using Markov Decision Processes (MDPs), we then describe a canonical generative model and the ensuing update equations that minimize free-energy. We then apply these equations to simulations of foraging in an environment; in which an agent learns the most efficient path to a pre-specified location. In some of those simulations, unbeknownst to the agent, the ‘desire paths’ emerge as a function of the activity of the agent (i.e. niche construction occurs). We will show how, depending on the relative inertia of the environment and agent, the joint agent-environment system moves to different attracting sets of jointly minimized free-energy.

## Highlights

• Free-energy is developed as a measure for the `fit’ between an agent and its niche.
• Simulations show how the behavior of an agent is shaped by the structure of its niche.
• Using computational methods, we show how niche-construction can improve the `fit’ between an agent and its environment.

## 1 Introduction

What does it mean to say that an agent is adapted to - or ‘fits’ - its environment? Strictly speaking, in evolutionary biology, fitness pertains only to the reproductive success of a phenotype over evolutionary time-scales (Orr, 2009). However, reproductive presupposes that an animal is sufficiently “adaptively fit”; to stay alive long enough to reproduce, given the statistical structure of its environment. On developmental time-scales, the animal comes to fit the environment by learning the statistics and dynamics of the ecological niche it inhabits. In other words, it acquires the skills to engage with the action possibilities available in its niche. On time-scales of perception and action, an organism improves its fit, or grip (Bruineberg and Rietveld, 2014), by selectively being sensitive to the action possibilities, or affordances (Gibson, 1979, Rietveld and Kiverstein, 2014) that are offered by the environment.

Agents can not only come to fit their environments, but environments can come to fit an agent, or a species. For example, earth worms change the structure and chemical composition of the soil they inhabit and as a consequence, inhabit radically different environments in which they are exposed to different selection pressures -compared a previously uninhabited piece of soil (Darwin, 1881, Odling-Smee et al., 2003). In evolutionary biology, the process by which an agent alters its own environment to increase its survival chances is better known as “niche construction” (Lewontin, 1983, Odling-Smee et al., 2003). This leads to a feedback mechanism in evolution, whereby a modification of the environment by members of a species alter the developmental trajectories of its members and the selection pressures working on its members.

In the niche construction literature, a distinction is made between *selective niche construction* and *developmental niche construction*. Selective niche construction pertains to the active modification of an environment so that the selection pressures on hereditary traits change as a result of these modifications. Developmental niche construction, on the other hand, pertains to the construction of ecological and social legacies that modify the learning process and development of an agent (Stotz, 2017). In this paper, we focus on developmental niche construction. An example of this form of niche construction is the so-called `desire path’: rushing on their way to work, people might cut the corner of the path through the park. While initially this might almost leave no trace, over time a path emerges, in turn attracting more agents to take the shortcut and underwrite the path's existence. Such `desire paths’^1^ are fascinating examples of developmental niche construction and their emergence is a key focus of this paper.

The aim of this paper is to discuss and model developmental niche construction in the context of active inference and the free-energy principle (Friston and Stephan, 2007). The free-energy principle is a principled and formal attempt to describe the ‘fit’ between an embodied agent and its niche, and to explain how agents perceive, act, learn, develop and structure their environment in order to optimize their fitness, or minimize their free-energy (Friston and Stephan, 2007, Friston et al., 2010). The free-energy principle pertains to the fitness of an agent in its environment over multiple time-scales, ranging from the optimization of neuronal and neuromuscular activity at the scale of milliseconds to the optimization of phenotypes over evolutionary timescales (Friston, 2011, Fig. 10).

We will apply the free-energy principle to an agent's active construction of a niche over the time-scales of action, perception, learning and development. We are therefore not directly concerned with *reproductive fitness* (the reproductive success of an agent) but rather with *adaptive fitness* (how well an agent is fairing in its interactions with the environment). The adaptive ‘fit’ between agent and environment is in this paper characterized by the information-theoretic quantity of (variational) free-energy.^2^

There are potentially many ways to model niche construction, using conceptual analysis, numerical analysis or formal models that vary in their form and assumptions: see (Creanza and Feldman, 2014, Krakauer et al., 2009, Laland et al., 1999, Lehmann, 2008) for some compelling examples. The modelling framework we use is somewhat unique in that it uses generic (variational) principles to model any self-organising system in terms of information theory or belief updating. The usual applications of this model have been largely restricted to behavioural and cognitive neuroscience; e.g., (Friston et al., 2017a, Friston et al., 2017b, Kaplan and Friston, 2018). Here, we apply exactly the same principles and model to niche construction – to implement an extended aspect of active inference (a.k.a., the free energy principle). The advantage of this is that one has a principled and generic framework has a well formulated objective function and comes equipped with some fairly detailed process theories; especially for phenotypic implementation at the neuronal level (Friston et al., 2017a, Friston et al., 2017b). Conceptually, this means one can cast niche construction as an inference process; thereby providing an interesting perspective on the circular causality that underlies niche construction.

The “fit” between the agent and its environment can be improved both by the agent coming to learn the structure of the environment and by the environment changing its structure in a way that better fits the agent. This gives rise to a continuous feedback loop, in which what the agent does changes the environment, which changes what the agent perceives, which changes the expectations of the agent, which in turn changes what the agent does (to change the environment). The interesting point here is that the minimum of free-energy is not (necessarily) at a point where the agent is maximally adapted to the statistics of a given environment, but can better be conceptualized as a stable point or, more generally, an attracting set of the *joint agent-environment system*.

The attracting set – on which an agent-environment system settles - will depend upon on the malleability of both the agent and the environment. In the limiting case of a malleable agent and a rigid environment, this amounts to learning. In the other limiting case of a rigid agent and a compliant environment, we find niche construction (making the world conform to one's expectations). In intermediate cases, both the agent and the environment are (somewhat) malleable. Importantly, as we will see later on in this paper, the malleability of the agent and the environment can be given a concise mathematical description in terms of the prior beliefs. These prior beliefs reflect the influence sensory evidence has on learning. In other words, they determine the ‘learning rate’ or ‘inertia’ of both the agent and the environment. These learning rates^3^ embody the evolutionary and developmental history of an agent (the stability of the niche an agent evolved in) and the type of environment involved.

In brief, the active inference formulation described below offers a symmetrical view of exchanges between agent and environment. The effect of the agent on the environment can be understood as the environment `learning’ about the agent through the accumulation of ecological legacies (Laland et al., 2016). This perspective is afforded by the basic structure of active inference that rests upon the coupling between a *generative process* (i.e., environment) and a *generative model* of that process (i.e., agent). The mutual adaptation between the process and model means that there is a common phenotypic space that is shared by the environment and agent. On this view, the environment acts upon the agent by supplying sensory signals and senses the agent through the agent's action. Mathematically, the environment accumulates evidence about the generative models of the agents to which it plays host. This symmetry plays out in a particular form, when we consider the confidence or precision placed in the prior beliefs of the environment and agent – and the effect the relative precisions have on the convergence or (generalized) synchronization that emerges as the agent and environment ‘get to know each other’.

In what follows, we will provide a general introduction to active inference and the free-energy principle. Using Markov Decision Processes (MDPs), we then describe a canonical generative model and the ensuing update equations that minimize free-energy. We then apply these equations to simulations of foraging in an environment; in which an agent learns the most efficient path to a pre-specified location. In some of those simulations, unbeknownst to the agent, the environment changes as a function of the activity of the agent (i.e. niche construction occurs). We will show how, depending on the relative inertia of the environment and agent, the joint agent-environment system moves to different attracting sets of jointly minimized free-energy.

## 2 The free-energy principle and active inference

The motivation for the free-energy principle is to provide a framework in which to treat self-organizing systems and their interactions with the environment. Below, we will briefly rehearse the arguments that lead from the desideratum of self-organization to the minimization of free-energy: for details, see Friston and Stephan, 2007, Friston, 2011 and, in more conceptual form, Bruineberg et al. (2016).

The starting point of the free-energy principle is the observation that living systems maintain their organization in precarious conditions. By precarious we mean that there are states an organism *could* occupy but at which the organism would lose its organization. Hence, if we consider a state space of all the situations an organism can be in (both viable and lethal) we will observe (by necessity) that there is a very low probability of finding an agent in the lethal parts of the state space and a high probability it occupies viable parts. Although which states are viable is dependent on the kind of animal one observes; namely, on their *characteristic states*.

We assume the agent has sensory states that register observations or outcomes $\tilde{o}$, where outcomes are a function of the state of the agent's environment, or hidden states, $\tilde{s}$. These states are called “hidden” because they are “shielded off” from internal states by observation states. For an adaptive agent, its sensory states support a probability distribution $P(\tilde{o})$ with high probability of being in some observation states, and low probability of being in others, where - in analogy with the hidden state - frequently occurring outcome states are associated with viable, characteristic states and very rare outcome states are associated with potentially lethal states (see Table 1 for notation, we will denote actual states in the environment with bold face $\tilde{s}$, and states the agent expects in the environment using normal script $\tilde{s}$). Given the distribution $P(\tilde{o})$, one can calculate the surprisal (unexpectedness) of a particular observation *o*: −lnP(o). Observations that are encountered often, or for a long time, will have low surprisal, while outcomes that are (almost) never observed will have very high surprisal.

**Table 1:** Glossary of variables and expressions.

	Expression	Description
$P(\tilde{o},\tilde{s},\pi ,\theta )$		*Generative model (agent):* joint probability of observations $\tilde{o}$, hidden states $\tilde{s}$, policies *π*, and parameters *θ*. Returns a sequence of actions $u_{t}=\pi \left(t\right).$
*o_τ_*	∈ {0, 1}	Outcomes and their posterior expectations
${\hat{o}}_{\tau }$	∈ [0, 1]	
$\tilde{o}$	$=(o_{1},\ldots .,o_{t})$	Sequences of outcomes until the current time point.
*s_τ_*	∈ {0, 1}	Inferred hidden states and their posterior expectations, conditioned on each policy.
${\hat{s}}_{\tau }^{\pi }$	∈ [0, 1]	
$\tilde{s}$	$=(s_{1},\ldots .,s_{T})$	Sequences of inferred hidden states until the end of the current trial.
${\hat{s}}_{\tau }$	$=\sum_{\pi }\pi \cdot {\hat{s}}_{\tau }^{\pi }$	Bayesian model average of hidden states over policies
*π*	$=\left(\pi_{1},\ldots ,\pi_{k}\right):\;\pi \in \left\{0,1\right\}$	Policies specifying action sequences and their posterior expectations.
$\hat{\pi }$	$=\left({\hat{\pi }}_{1},\ldots ,{\hat{\pi }}_{k}\right):\;\hat{\pi }\in \left[0,1\right]$	
*θ*	=(A,B,C,D)	Parameters of the generative model
*A*_*i, j*_	$=P(o_{t}=i|s_{t}=j)$	Likelihood matrix mapping from inferred hidden state *j* to an expected observation *i* and its logarithm.
$\bar{A_{i,j}}$	$=\ln A_{i,j}=\psi \left(\alpha_{i,j}\right)-\psi \left(\alpha_{0,j}\right)$	
*α*_*i, j*_	∈ *R*_ > 0_	The parameters of the agent's prior (Dirichlet) distribution for an observation *i* at location *j*.
*α*_0, *j*_	$=\sum_{i}\alpha_{i,j}$	Sum of concentration parameters over outcomes at a particular location.
$B_{i,j,t}^{\pi }$	$=P(s_{i,t+1}|s_{j,t},\pi )$	Transition probability for hidden states under each action prescribed by a policy at a particular time and its logarithm.
${\bar{B}}_{i,j,t}^{\pi }$	$=\ln B_{\tau }^{\pi }$	
*C*_*i, τ*_	$=-\ln P\left(o_{i,\tau }\right)↔P\left(o_{i,\tau }\right)=-\sigma \left(C_{i,\tau }\right)$	Logarithm of prior preference over outcomes or utility.
*D_j_*	$=P(s_{j,t=0})$	Prior expectation of the hidden state at the beginning of each trial.
*F_π_*	$=F\left(\pi \right)=\sum_{\tau }F\left(\pi ,\tau \right)\in R$	Variational free energy for each policy.
*G_π_*	$=G\left(\pi \right)=\sum_{\tau }G\left(\pi ,\tau \right)\in R$	Expected free energy for each policy.
*H*	$=-\sum_{k}A_{kl}A_{lk}$	Vector encoding the entropy or ambiguity over outcomes for each hidden state.
*ψ*(*α*)	$=\partial_{\alpha }\ln\Gamma \left(\alpha \right)$	Digamma function or derivative of the log gamma function.^a^
*W*	$=\frac{1}{a_{0}}-\frac{1}{a}$	A matrix encoding the uncertainty about parameters, for each combination of outcomes and hidden states. This represents the contribution these parameters make to the complexity (i.e. the expected difference between the logs of the posterior and prior parameters).
$P(\tilde{o},\tilde{s},\tilde{u},\theta )$		*Generative process (environment):* joint probability of observations $\tilde{o}$, hidden states $\tilde{s}$, actions ***u***, and parameters ***θ***. Generates observations: $o_{t}=As_{t}.$
***θ***	=(A,B,C,D)	Parameters of the generative process
***s***_***τ***_	∈ {0, 1}	Actual hidden state, (analogous notation for posterior and sequences).
***u***_***t***_	=π(t)	Action or control variables
$\tilde{u}$	$=(u_{1},\ldots .,u_{T})$	Sequences of action or control variables until the end of the current trial.
***A***_***i, j***_	$=P(o_{t}=i|s_{t}=j)$	Likelihood matrix mapping from environmental hidden state *j* to observation *i* and its logarithm (analogous notation for concentration parameters).
$\bar{A_{i,j}}$	$=\ln A_{i,j}=\psi \left(\alpha_{i,j}\right)-\psi \left(\alpha_{0,j}\right)$	
***α***_***i, j***_	∈ *R*_ > 0_	The parameters of the environmental (Dirichlet) distribution for an observation *i* at location *j*.
***α***_0, ***j***_	$=\sum_{i}\alpha_{i,j}$	Sum of concentration parameters over outcomes at a particular location.

a The derivation of the belief updating using digamma functions can be found in the appendix of (Friston et al., 2016), which also provides a more intuitive interpretation in terms of (neuronal) plasticity.

One expects a certain degree of recurrence in the states one finds any creature in. Take, for example, a rabbit: the typical situations a rabbit finds itself in might be eating, sheltering, sleeping, mating etc. It will repeatedly encounter these states multiple times throughout its life. Under mild^4^ assumptions, the frequency with which we expect to find the rabbit in a particular state over time is equal to the probability of finding the rabbit in that particular state at *any* point in time. This implies that the average surprisal over time is equal to the expected surprisal at any point in time, or mathematically:^5^

$$\sum_{s}-P\left(s\right)\ln P\left(s\right)=\sum_{t}^{T}-\frac{1}{T}\ln P\left(s_{t}\right)$$

### 2.1 Free-energy and self-organization

So far, we have adopted a descriptive point of view, starting from an adaptive agent. We can now turn from the descriptive statement - that adaptive agents occupy a restricted (characteristic) part of the state space with high probability - to the normative statement that in order to *be* adaptive, it is sufficient for the agent to occupy a characteristic part of the state space, which (by definition) must be compatible with the characteristic states of the agent in question. For example, the human body performs best at a core body temperature around 37 °C. When measuring the temperature of a human, one expects to measure a core body temperature around 37 °C, while measuring a body temperature of 29 °C or 41 °C would be very surprising and indicative of a threat to the viability of the agent. For adaptive temperature regulation then, it is sufficient to minimize the surprisal of observational states $\tilde{o}$ with respect to a probability distribution $P(\tilde{o})$^6^ peaking at those temperature values that are characteristic of human bodies.

The observational states $\tilde{o}$ and the probability distribution $P(\tilde{o})$ serve to make the surprisal of an observation $-\ln P(\tilde{o})$ accessible to the agent. The ecologically relevant question for the agent is however how to minimize the surprisal of observations. Minimization of surprisal can only be achieved through action, be it by acting on the world (for example by moving into the shade) or changing the body (for example by activating sweat glands). That is to say, the agent needs to predict how actions ***u*** impact on observational states *o*. More often than not, the impact of control or active states ***u*** will be mediated by the hidden state of the environment ***s***: the action that reduces surprisal of temperature sensors depends on where the agent can find shade. Moreover, in many cases, surprising observational states can only be avoided by eluding particular hidden states in the environment pre-emptively. For example, a mouse can avoid being eaten by a bird of prey (a highly surprising state of affairs for a living mouse), by avoiding hidden states in which a bird of prey can see it. In turn, the diving bird causes a particular observation in the mouse (a fleeting shadow, i.e. a sudden decrease in light intensity on its sensory receptors). The mouse therefore *needs* to treat the observation generated by a bird of prey as an unlikely state and avoid it by acting. Whether a particular, surprising, observation is encountered therefore depends upon the hidden states of the world that cause observations Crucially, in order to minimize the surprisal of observations, the agent also needs to be able to predict the consequences of its actions on the environment.

The surprisal of observations is therefore the marginal distribution of the joint probability of observations, marginalized over hidden states and policies the agent pursues:

$$-\ln P\left(\tilde{o}\right)=-\ln\sum_{s,u,\theta }P\left(\tilde{o},\tilde{s},\tilde{u},\theta \right)$$

The probability distribution $P(\tilde{o},\tilde{s},\tilde{u},\theta )$ is known as the *generative process* (where ***θ*** represents a set of parameters), denoting the actual causal, or correlational, structure between action states $\tilde{u}$, hidden states $\tilde{s}$, and observation states $\tilde{o}$, parametrized by ***θ***. Importantly, the agent only has access to a series of observations $\tilde{o}$ and not to hidden states $\tilde{s}$ and actions $\tilde{u}$. This means it cannot perform the marginalization above; instead we assume the agent uses a *generative model*$\;P(\tilde{o},\tilde{s},\pi ,\theta )$, denoting the agent's expectations about the causal structure of the environment (generative process) and the policies it pursues.

**Fig. 1:**
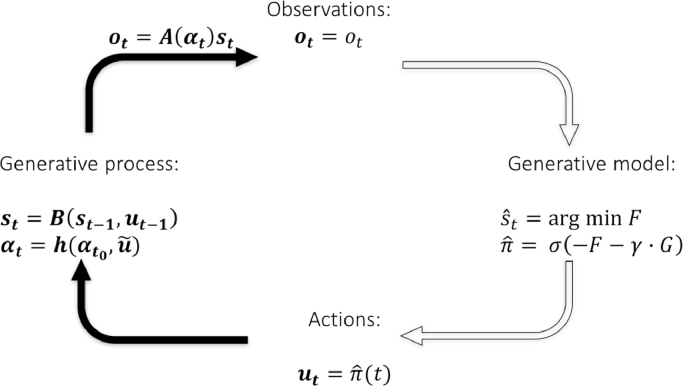
The generative process and model and their points of contact: The generative process pertains to the causal structure of the world that generates observations for the agent, while the generative model pertains to how the agent expects the observations to be generated. A hidden state in the environment *s_t_* delivers a particular observation *o_t_* to the agent. The agent then infers the most likely state of the environment (by minimizing variational free-energy) and uses its posterior expectations about hidden states to form a posterior over policies. These policies specify actions that change the state (and parameters) of the environment.

We can now discuss the implications of this separation between the *generative process* and the *generative model*. The generative process pertains to the actual structure of the world that generates observations for the agent. In contrast, the generative model pertains to how the agent expects the observations to be generated. The agent will intervene in the world under the assumption that its generative model is close^7^ to the generative process. If the generative process is initially very different to the model, the interventions of the agent change the process to more closely resemble the model. The notion that the generative model and process should resemble one another relates to the ‘Good Regulator Theorem’ of Conant and Ashby (1970). In our context, this theorem implies that the capacity to regulate one's econiche depends upon how good a model one is of that niche. That is to say, the structure captured in the generative model will pertain to ecologically relevant aspects of the environment (Baltieri et al., 2017). The generative model and process meet at two places: the environment is causing the observation states of the agent, and actions are sampled from a distribution over policies, selected by the agent under its generative model (see Fig. 1).

Note that, from the perspective of the agent, the agent uses its generative model to evaluate the surprisal (or negative log evidence) of observations:

$$-\ln P\left(\tilde{o}\right)=-\ln\sum_{s,\pi ,\theta }P\left(\tilde{o},\tilde{s},\pi ,\theta \right)$$

However, although the agent has access to all the variables in the above equation, this marginalization is analytically intractable; so the minimization of surprisal is not possible directly. Instead, one can consider an upper bound on surprisal that can be evaluated and subsequently minimized; thereby explaining surprisal minimizing exchange with the environment in a way that can be plausibly instantiated in a living creature.

One can construct this upper bound by adding an arbitrary distribution $Q(\tilde{s},\pi ,\theta )$ to the surprisal term and using the definition of the expectation or expected value $E_{q(x)}\left[x\right]=\sum_{x}q\left(x\right)\cdot x$:

$$\begin{array}{ccc} -\ln P\left(\tilde{o}\right) & = & -\ln\sum_{s,\pi ,\theta }Q\left(\tilde{s},\pi ,\theta \right)\frac{P\left(\tilde{o},\tilde{s},\pi ,\theta \right)}{Q\left(\tilde{s},\pi ,\theta \right)}\\ & = & -\ln E_{Q\left(\tilde{s},\pi ,\theta \right)}\left[\;\frac{P\left(\tilde{o},\tilde{s},\pi ,\theta \right)}{Q\left(\tilde{s},\pi ,\theta \right)}\right] \end{array}$$

Using Jensen's inequality (following from the concavity of the log function), we then have the following inequality:

$$\begin{array}{ccc} -\ln P\left(\tilde{o}\right) & = & -\ln E_{Q\left(\tilde{s},\pi ,\theta \right)}\left[\;\frac{P\left(\tilde{o},\tilde{s},\pi ,\theta \right)}{Q\left(\tilde{s},\pi ,\theta \right)}\right]\\ & \leq & -E_{Q\left(\tilde{s},\pi ,\theta \right)}\left[\ln\left(\;\frac{P\left(\tilde{o},\tilde{s},\pi ,\theta \right)}{Q\left(\tilde{s},\pi ,\theta \right)}\right)\right]=F \end{array}$$

The term on the right-hand side of the equation - the free-energy *F* - is therefore an upper bound on the term on the left-hand side of the equation, the surprisal of observations. In short, minimizing free-energy implicitly minimizes surprisal.

### 2.2 Free-energy and variational inference

The question then is how the minimization of free-energy can be achieved, and what this optimization entails. We have defined free-energy in terms of a generative model $P(\tilde{o},\tilde{s},\pi ,\theta )\;$and an arbitrary variational distribution $Q(\tilde{s},\pi ,\theta )$. The free-energy can be written in several forms to show what its minimization entails, specifically:

$$F\left(\tilde{s},\pi ,\theta \right)=\underset{divergence}{\underset{︸}{D_{KL}\left[Q\left(\tilde{s},\pi ,\theta \right)∥P\left(\tilde{s},\pi ,\theta |\tilde{o}\right)\right]}}\;-\underset{\log\;evidence}{\underset{︸}{\ln P(\tilde{o})}}$$

This formulation shows the dependency of the free-energy on beliefs about the hidden states implicit in the variational distribution. Since the negative log evidence, or surprisal, does not depend on $Q(\tilde{s},\pi ,\theta )$, optimizing the variational distribution to minimize free-energy means that the divergence from the posterior $p(\tilde{s},\pi ,\theta |\tilde{o})$ is minimized. This makes $Q(\tilde{s},\pi ,\theta )$ an approximate posterior, i.e., the closest approximation of the true posterior $P(\tilde{s},\pi ,\theta |\tilde{o})$. This highlights the relationship between free-energy minimization and theories of perception as Bayesian inference (Gregory, 1980). Furthermore, since the KL-divergence is always greater than zero, minimizing free energy makes it a tight upper bound on surprisal.

Whether the exact minimization of free-energy is feasible depends on the generative process and generative model. Typically, simplifying assumptions need to be made about the form of the variational distribution, resulting in approximate rather than exact inference. The most ubiquitous assumption about the variational distribution is that it can be factorized into marginals. This is known as the mean field approximation (Opper and Saad, 2001). The only parameters *θ* that will vary in this paper are the parameters of an observation matrix *A*⊂*θ* and we can deal with a variational distribution of the form:

$$Q\left(\tilde{s},\pi ,A\right)=Q\left(\pi \right)Q\left(A\right)\prod_{t}^{T}Q(s_{t}\left|\pi \right)$$

The challenge now is to find the approximate posterior $\tilde{Q}$ that minimizes free-energy given a series of observations $\tilde{o}$ and the generative model $P(\tilde{o},\tilde{s},\pi ,\theta )$. In other words, we want to find those $\tilde{Q}$ such that:

$$Q\left(\tilde{s},\pi ,A\right)=\text{arg}\;\min_{Q}F\approx P\left(\tilde{s},\pi ,A|\tilde{o}\right)$$

This will provide update equations that formalize the exchange between the agent and its environment that is consistent with its existence, through a variational process of self-organisation. Due to the way the variational distribution is factorized, each factor can be optimized separately. The specific update equations specified in the next section are obtained by taking the functional derivative of the free-energy with respect to each factor and solving for zero. We can then construct a differential equation whose fixed point coincides with this solution, i.e. the minimum of free-energy. The result is a set of self-consistent update equations that converge upon the minimum of free-energy (see Appendix B and Friston et al., 2016a, Friston et al., 2016b). Although not relevant for the current treatment, these equations have a lot of biological plausibility in terms of neuronal processes – and indeed non-neuronal processes involving cellular interactions: for further discussion, see (Friston et al., 2017a, Friston et al., 2017b). In short, if these variational constructs are the only way to solve a problem that is necessary to exist in a changing world, we can plausibly assume that evolution uses these constructs: more precisely, evolution is itself a form of variational free energy minimization (see discussion).

### 2.3 Adaptive action and expected free-energy

Policies, or sequences of actions, do not alter the current observations, but only observations in the future. This suggests that the dynamics we are trying to characterize must be based upon generative models of the future. Furthermore, this means that an agent selects those policies that it expects will make it keep minimizing free-energy in the future. This requires us to define an additional quantity, *expected* free-energy *G*, to ensure the agent acts so as to minimize the expected surprisal under a particular policy (i.e., pursue uncertainty-resolving, information-seeking policies that exploit epistemic affordances (Kiverstein et al., 2017) in their econiche). Above, we have defined the free-energy as:

$$F=E_{Q\left(\tilde{s},\pi ,\theta \right)}\left[\ln Q\left(\tilde{s},\pi ,\theta \right)-\ln P\left(\tilde{o},\tilde{s},\pi ,\theta \right)\right]$$

In analogy with the variational free-energy, we can now define an expected free-energy under a particular policy *π*:

$$G\left(\pi \right)=\sum_{\tau }G\left(\pi ,\tau \right)$$

$$G\left(\pi ,\;\tau \right)=E_{\tilde{Q}}\left[\ln\;Q\left(s_{\tau }|\pi \right)-\ln P\left(s_{\tau },o_{\tau }|\tilde{o},\pi \right)\right]$$

where $\tilde{Q}=Q\left(o_{\tau },s_{\tau }|\pi \right)=P\left(o_{\tau }|s_{\tau }\right)Q\left(s_{\tau }|\pi \right)$. In other words, the expectation is taken under a counterfactual distribution $\tilde{Q}$ over hidden states and yet to be observed outcomes (and not over hidden states and policies, as was the case for the variational free-energy). Rearranging this expected free energy gives (see Appendix):

$$G\left(\pi ,\;\tau \right)=D_{KL}\left[Q\left(o_{\tau }|\pi \right)P\left(o_{\tau }\right)\right]+E_{Q\left(s_{\tau }|\pi \right)}H\left[P\left(o_{\tau }|s_{\tau }\right)\right]$$

Here, the second term is called ambiguity and reflects the expected uncertainty about outcomes, conditioned upon hidden states. The first term is the divergence between prior (i.e., preferred or characteristic) outcomes and the outcomes expected under a particular policy. This *Bayesian risk* or expected cost is the smallest for a policy that brings about observations that are closest to preferred observations. We can operationalise this sort of policy selection with a prior over policies that can be expressed as a softmax function of expected free-energy:

P(π)=σ(−G(π))

In short, the agent selects policies that it expects will minimize the free-energy of future observations (see Appendix A). This is equivalent to minimizing Bayesian risk and resolving ambiguity.

So what does the minimization of free-energy entail in different contexts? In the limiting case of *perceptual inference* (where the agent cannot change the sensory array it is exposed to), free-energy is minimized by finding the hidden states $\tilde{s}$ that most likely generated observed sensory states $\tilde{o}$, under the agent's generative model of how they co-occur. This makes the recognition distribution $Q(\tilde{s})$ an approximate conditional distribution $P(\tilde{s}|\tilde{o})$. Here, the expected hidden states are the parameters of the variational distribution, which are generally considered to be internal states of the agent (e.g., neuronal activity).

When actions are allowed, but the agent has no preferences for particular states (*active inference without preferences*), free-energy is minimized by finding the hidden states $\tilde{s}$ that most likely generated observed sensory states $\tilde{o}$ and those actions are selected that minimize the ambiguity of observations given hidden states *P*(*o_t_*|*s_t_*). This puts both action and perception in the fame of hypothesis-testing, or optimizing the Bayesian model evidence of an agent's model of its environment, licensing a Helmholtzian interpretation of the activity of the brain (Friston et al., 2012).

However, when the agent is equipped with preferred sensory observations (*active inference with preferences*), the picture changes profoundly (Bruineberg et al., 2016). Besides finding the hidden states $\tilde{s}$ that most likely generated observed sensory states $\tilde{o}$ the goal is also to select those actions that bring about preferred outcomes; enabling it to elude surprising states of affairs. To give an intuitive example, the agent's current sensations might best be explained by the conjecture that he is standing under a shower that is too hot - a fairly unambiguous signal. But, if all is well, standing under un uncomfortably hot shower is itself a highly surprising event. He will therefore reach for the tap to reduce the temperature and seek sensory evidence from the world that he is standing under a comfortable shower, which is unsurprising. In other words, the agent does not continue to infer the hidden cause of its original surprising observations (i.e. that it is a very hot shower), but rather *intervenes in the world* so as to bring about preferred states that fit his prior expectations about the sorts of sensations he expects to encounter.

Active inference *with* preferences therefore changes the epistemic pattern the agent engages in. Rather than, analogous to a rigorous scientist, inferring the causal structure of the world by probing it and observing the resulting data, the agent acts like a *crooked* scientist, expecting the world to behave in a particular kind of way and through changing the world, ensures that those expectations come true (Bruineberg et al., 2016).

This changes the interpretation of free-energy minimization: in active inference *without prior* preferences, the minimum of free-energy coincides with an agent that comes to infer the hidden structure of the world. In active inference *with* preferences, the minimum of free-energy is attained when sensations are generated by characteristic or preferred states that are realized through action (Friston, 2011).^8^ In this latter way, crucially, the free-energy principle provides a common currency for both epistemics (finding out about the state of the world) and value (engaging with the world to seek out preferred outcomes). Agents are adaptive if they expect to be in states they characteristically thrive in and, through action, make those expectations come true.

What we have shown in this section is that what exactly *is* the minimum of free-energy differs depending on the assumption one makes about the nature of the agent and the task at hand: it coincides with an epistemic fit if one assumes perceptual inference and active inference *without* preferences, and it coincides an epistemically enriched value-based, pragmatic fit in the case of active inference *with* preferences. In the context of certain perceptual decision-making experiments carried out in a lab, such as the widely used random-dot motion task (e.g., Ball and Sekuler, 1982, Newsome and Pare, 1988) it might make sense to treat a rational agent as not having intrinsic preferences for a direction of motion. However, in an ecological setting, what matters is not just what the cause of the current sensory input is, but to be sensitive to the implicit pragmatic and epistemic affordances that enable the selection of actions that lead to preferred, or characteristic, sensory exchanges.

Because the prior preferences ensure that creatures act in ways that minimize expected free-energy, if they have the right sort of generative model, agents will, in acting, obtain the sensory evidence they expect. Incidentally, the addition of expected free-energy elegantly solves the dark-room problem (Friston et al., 2012): although being in a dark room makes sensory input very predictable, it is not the kind of situation a human phenotype expects to find itself in for long periods (although a bat might). The agent therefore treats these observations as surprising and tends to more characteristic sensory exchanges with the environment. This concludes our formal description of active (embodied) inference and the ensuing sort of self-organisation that emerges from it. We now turn to simulations to illustrate that free-energy minimization cuts both ways in an agent-environment exchange.

## 3 Simulation of niche construction

So far, we have addressed the motivation for, and derivation of, the free-energy principle and how actions underwrite the minimization of expected free-energy. We now turn to simulations of niche-construction using a free-energy minimizing agent. In order to do this, we need to make specific assumptions about the structure and parameters of the generative model that is constituted by the agent – and the generative process in the econiche. In brief, we will use a very simple model of the world that can be thought of as a maze that can be explored. Crucially, the very act of moving through the maze changes its state; thereby introducing a circular causality between the environment (i.e., maze) and a synthetic creature (i.e., agent), who traverses the environment, in search of some preferred location or goal.

**Fig. 2:**
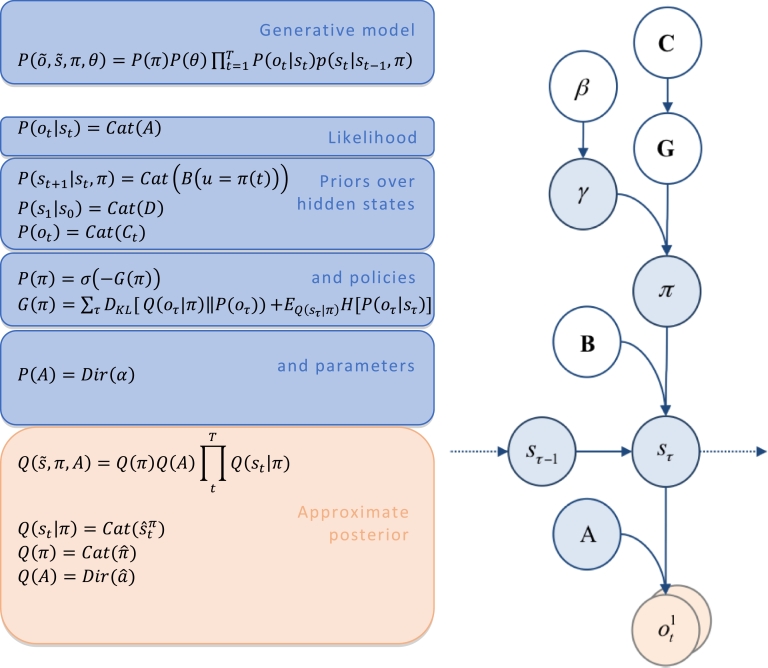
**Generative model and (approximate) posterior. Left panel:** A generative model is the joint probability of outcomes $\tilde{o}$, hidden states $\tilde{s}$, policies *π* and parameters *θ*: see top equation. The model is expressed in terms of the *likelihood* of an observation *o_t_* given a hidden state *s_t_*, and *priors* over hidden states: see second equation. In Markov decision processes, the likelihood is specified by an array *A*, parameterized by concentration parameters *α*. As described in Table 3, this array comprises columns of concentration parameters (of a Dirichlet distribution). These can be thought of as the number of times a particular outcome has been encountered under the hidden state associated with that column. The expected likelihood of the corresponding outcome than simply entails normalising the concentration parameters so that the sum to 1. The empirical priors over hidden states depend on the probability of hidden states at the previous time-step conditioned upon an action *u* (determined by policies *π*), these probabilistic transitions are specified by matrix *B*. The important aspect of this generative model is that the priors over policies *P*(*π*) are a function of expected free-energy *G*(*π*). That is to say, *a priori* the agent expects itself to select those policies that minimize expected free-energy *G*(*π*) (by minimizing its path integral $\sum_{\tau }G\left(\pi ,\;\tau \right)$). See the main text and Table 1 for a detailed explanation of the variables. In variational Bayesian inversion, one has to specify the form of an approximate posterior distribution, which is provided in the lower panel. This particular form uses a mean field approximation, in which posterior beliefs are approximated by the product of marginal distributions *Q*(*s_t_*|*π*) over unknown quantities. Here, a mean field approximation is applied to both posterior beliefs at different points in time *Q*(*s_t_*|*π*), policies *Q*(*π*), parameters *Q*(*A*) and precision *Q*(*γ*). **Right panel:** This Bayesian graph represents the conditional dependencies that constitute the generative model. Blue circles are random variables that need to be inferred, while orange denotes observable outcomes. An arrow between circles denotes a conditional dependency, while the lack of an arrow denotes a conditional independency, which allows the factorization of the generative model, as specified on the left panel.

To build this simulation, we will assume some specific conditional independencies that render the generative model a so-called Markov Decision Process (MDP). The main two features of Markov decision processes are i.) that observations at a particular time *o_t_* depend only on the current hidden state *s_t_*, and 2.) the probability of a hidden state $s_{t+1}$depends only on the previous hidden state *s_t_* and the policy *π*(*t*) (see Fig. 2, right panel). Each of the probabilistic mappings or transitions is parameterized by a distribution matrix (Fig. 2, left hand side). The outcome or likelihood matrix is given by *A*, where $A_{ij}=P\left(o_{t}=i|s_{t}=j\right)$. The probability transition matrix of hidden states over time is given by *B*, where $B_{ij}\left(u\right)=P\left(s_{t+1}=i|s_{t}=j,\;\pi \left(t\right)=u\right)$. *C* denotes prior (preferred) beliefs about outcomes *P*(*o_t_*) and *D* denotes beliefs about the initial states at t = 1. These conditional probabilities can be seen in Fig. 2. As above, we define the variational distribution as:

$$Q\left(\tilde{s},\pi ,A\right)=Q\left(\pi \right)Q\left(A\right)\prod_{t}^{T}Q(s_{t}\left|\pi \right)$$

**Fig. 3:**
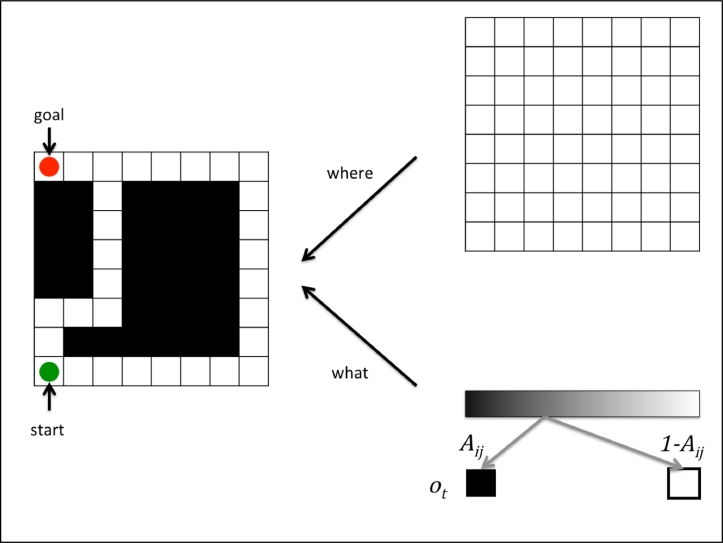
**The layout of the environment:** The agent's environment comprises an 8 × 8 grid. At each square the agent observes its current location (‘where’ hidden state) and either an ‘open’ or ‘closed’ state (‘what’ hidden state). The mapping from hidden states to observations in the ‘where’ modality is direct (i.e., one-to-one). In the ‘what’ modality, the statistics of the environment are given by the **A**-matrix. An outcome is generated probabilistically based on the elements of the A-matrix at a particular location. The agent starts at the left bottom corner of the grid (green circle) and needs to go to the left top corner (red circle). (For interpretation of the references to color in this figure legend, the reader is referred to the web version of this article.)

In what follows, we describe the particular form of the generative model – in terms of its parameters, hidden states and policies – that will be used in the remainder of this paper. An agent starts at a specified location (Fig. 3- green circle) on an 8 × 8 grid and is equipped with a prior belief it will reach a goal location (Fig. 3– red circle) within a number of time steps, (preferably) without treading on `closed’ (black) squares. The agent's visual input is limited, in the sense that it can only see whether its current location is open (white) or closed (black). This means that, in the absence of prior knowledge, an agent needs to visit a location in order to gather information about it.

Each trial comprises several epochs. At each epoch, the agent observes its current position, carries out an action: moving up, down, left, right, or stay, and samples its new position. A trial is complete after a pre-specified number of time steps. In addition to visual input, we also equip the agent with positional information; namely its current location. This means that there are two outcome modalities (*o_t_*): *what* (open/white vs. closed/black) and *where* (one of 64 possible locations) (see Fig. 3). The generative model of these outcomes is simple: the hidden states (*s_t_*): correspond to the 64 positions. The likelihood mapping for the *where-*modality corresponds to an identity matrix, returning the veridical location for each hidden state. For the *what*-modality, the likelihood matrix specifies the probability of observing an open versus a closed state: $A_{ij}^{what}=P\left(o_{t}=white|s_{t}\right)$, parametrized by concentration parameters (see below). The (empirical) probability transitions are encoded in five matrices (corresponding to the 5 policies of the agent: $B_{ij}^{\pi }=P\left(s_{t+1}=i|s_{t}=j,\pi \right)$. These matrices move the hidden (*where*) states to the appropriate neighbouring location given the policy. The *D* vector designates the true starting location of the agent. Prior beliefs over allowable policies depend on expected free-energy *G*(*π*), which depends on prior preferences, or costs, over outcomes *C* (see below). When the parameters are unknown, as is the case for *A*, the parameters are modeled using Dirichlet distributions over the corresponding model parameters. The Dirichlet form is chosen because it is the conjugate prior for the categorical distributions that are used in this paper. The distribution is parameterised by a vector of concentration parameters (*α*) (see Table 3). Based on the particular generative model, one can derive the update equations (Table 2) that underwrite the minimization of free-energy (see Appendix B and Friston et al., 2015).

**Table 2:** Variational update equations.

Variational updates for the parameters (i.e. expectations) of the approximate posterior distribution		
*Perception and state-estimation*		
$s_{t}^{\pi }$	=	$\sigma (v_{t}^{\pi })$
${\dot{v}}_{t}^{\pi }$	=	$\bar{A}\Delta o_{t}+\bar{B_{t-1}^{\pi }}s_{t-1}^{\pi }-\bar{B_{t}^{\pi }}\cdot s_{t+1}^{\pi }-v_{t}^{\pi }$
$o_{t}^{\pi }$	=	$A\;s_{t}^{\pi }$
*Evaluation and policy selection*		
*π*	=	σ(−F−G)
*F_π_*	=	$\sum_{t}s_{t}^{\pi }\cdot \left(\ln s_{t}^{\pi }-\bar{B_{t-1}^{\pi }}s_{t-1}^{\pi }\right)-\sum_{t}s_{t}^{\pi }\cdot \bar{A}\cdot \;o_{t}$
*G_π_*	=	$\sum_{t}o_{t}^{\pi }\cdot \left(W\cdot s_{t}^{\pi }+\ln o_{t}^{\pi }+C_{t}\right)+H\cdot s_{t}^{\pi }$
*Precision and confidence*		
$\hat{\beta }$	=	$\left(\pi -\pi_{0}\right)\cdot G+\beta -\hat{\beta }$
*π*_0_	=	σ(−G)
*Bayesian model averaging and learning*		
*E_Q_*[*s_t_*]	=	$\sum_{\pi }\pi_{\;\pi }\cdot s_{t}^{\pi }$
$\ln{\hat{A}}_{t}$	=	$\psi \left(\alpha \right)-\psi \left(\alpha_{0}\right)$
${\hat{a}}_{t}$	=	$a_{t}+o_{t}⊗s_{t}$
*Change of the environment*		
$\ln{\hat{A}}_{t}$	=	$\psi \left(\alpha \right)-\psi \left(\alpha_{0}\right)$
${\hat{a}}_{t}$	=	$a_{t}+\left[\begin{array}{c} 1\\ 0 \end{array}\right]⊗s_{t-\Delta }$
*Action selection*		
$u_{t^{\prime }}$	=	$\max_{u}\pi \cdot \left[\pi (t)=u\right]$

### 3.1 Preferred outcomes and prior costs

The problem the agent faces is twofold. First, we want the agent to move from its start location to its target location; however, it can only see its current location and is only able to plan one move ahead. Second, the agent does not like treading on black (closed) squares, but at least initially, does not know which squares are black and which are white. Its job is then to find its way to the target location while avoiding black squares. The *A* matrix contains the agent's prior beliefs or preferences about outcomes in both modalities – *what* and *where*. At each epoch, the agent updates its prior beliefs based upon what it has come to know about the environment and selects its actions accordingly. In the current simulation, the agent's preferences or prior beliefs are that it will move towards a target location without transgressing into black squares. The subtle issue here is that the agent needs to select a policy that brings it closer to its goal state (taking into account what it knows about the layout of the environment) without performing an exhaustive search or planning into the far ahead future.

Intuitively, the agent's preferences can be understood in the following way: at each epoch, the agent expects to occupy locations that are not black, within the reach of its policies *and* are most easily accessible from the *target* location. Given that the agent's preferences are reconfigured after each epoch, the agent will inevitably end up at its target location. More formally, the expected cost (i.e. negative preference) of a sensory outcome at a future time *τ* can be described in the following way:

$$C_{\tau }=-\ln p\left(o_{\tau }\right)=\ln\left(\left[\exp\left(T\right)s_{1}<e^{-3}\right]+e^{-32}\right)-\ln\exp\left(T\right)s_{T}$$

Where:

$$T_{ij}=\left\{ \begin{array}{cc} -\sum_{i\neq j}T_{ij} & i=j\\ A_{i} & \exists u:B_{ij}^{u}>0\\ 0 & \text{otherwise} \end{array}\right.$$

Although the first term might look complicated, it just corresponds to a prior cost (of −32) whenever the condition in square brackets is not met, and zero otherwise. In other words, it assigns a high cost to any location that is occupied with a small probability when starting from the initial location *s*_1_. The second term corresponds to the (negative) log probability a given state is occupied when starting from the target location (*s_T_*), favoring states that are occupied with high probability. Prior beliefs about transitions are encoded in a `diffusion’ matrix exp (*T*). As noted in (Kaplan and Friston, 2018) the form of these priors is somewhat arbitrary but fairly intuitive. In brief, the graph Laplacian (*T*) allows us to express prior beliefs about preferred locations in terms of the probability of being in a particular place. Heuristically, the graph Laplacian models the dispersion of this probability – when moving in every allowable direction – as time progresses. If we combine this probability with the equivalent dispersion of probability mass from the goal location, their intersection identifies a plausible (preferred) location that can be accessed from the current location – and provides access to the goal.

The details of this particular prior cost function do not matter too much– they just serve to model preferences that lead to goal-directed behaviour under constraints and uncertainty. We have used these priors previously to simulate foraging in mazes (Kaplan and Friston, 2018). Here, we use the same setup but generalized to include an effect of navigating through the maze on the maze itself [Matlab code and demo routines detailing this generative model of spatial navigation are available in the **DEM Toolbox** of the **SPM open source software**: http://www.fil.ion.ucl.ac.uk/spm/]

### 3.2 Learning and the likelihood matrix

**Fig. 4:**
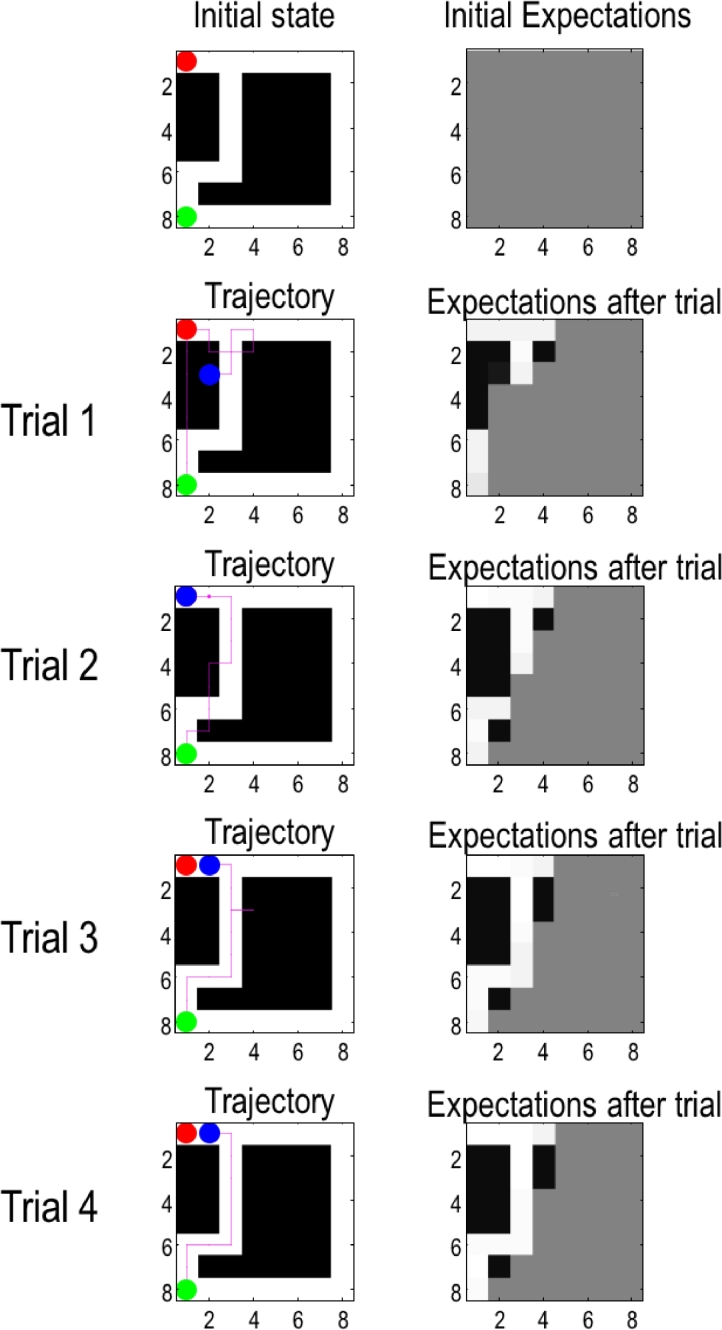
**Exemplar trials:** The left column shows the layout of the environment (**A**-matrix) and the right column shows the agent's expectations about the environment (A-matrix). The rows show the starting condition and the location after each trial. The green, red and blue circles designate the starting, target and final position respectively. The red-dotted line shows the agent's trajectory at other moves within a trial. In this and all subsequent examples, each trial comprised 16 moves. This figure illustrates four consecutive trials and consequent changes in the likelihood matrices that constitute the generative process (i.e. environment) and model (i.e. agent).

Although the graph Laplacian provides the agent with prior preferences (i.e., costs *C_τ_*), these are not the only factors underlying policy selection. The expected free-energy also contains an ambiguity term (see above and Appendix A) that is minimized when agents minimize the uncertainty of observations afforded by a particular location. This implies that the agent expects to explore its environment, even when this exploration does not bring it closer to its target state. This can be seen in Fig. 4, which shows the results of the simulation of successive trials. In the absence of any accumulated knowledge about the environment, the agent heads straight to its target state and then (rather than stay there) explores the local environment. In the next trial, the agent heads to its target state, while avoiding those locations that it now knows are closed. In the third trial, the agent has found the shortest (open) path to its target state, but still explores its surrounding, whenever in its vicinity ambiguity can be reduced. In trial four, and thereafter, the agent follows its “well trodden” and unambiguous white path.

At the beginning of a series of trials, the agent is initially naïve about the structure of the maze. This naivety can be quantified by equipping the agent with priors parameterized by Dirichlet distributions. The underlying concentration parameters of this prior can be thought of as the number of observations (or pseudo-observations) of a particular outcome the agent has already made before the start of a trial. In our case, the agent has separate concentration parameters for each outcome at each location. There are two relevant dimensions for the set of concentration parameters at a particular location: their absolute and their relative size. When the absolute size of the concentration parameters is low, the agent learns the hidden state (open or closed) of a location after one observation. When the concentration parameters – reporting the number of times open or closed outcomes have been experienced – are high, the agent needs many more observations to be convinced a state is open or closed (see Table 3). In short, the concentration parameters determine both the prior expectations about the world and the confidence placed in those expectations. This confidence or precision determines the impact of further evidence, which decreases with greater confidence.

**Table 3:** **Updating of concentration parameters –** Prior expectations about the layout of the environment are given by a Dirichlet distribution, which is parameterized by concentration parameters *α_white_* and *α_black_*. The agent's prior expectation about the state of the environment can be expressed in terms of the (relative value of the) concentration parameters. Concentration parameters are updated in proportion to the number of observations of a particular outcome.

**Fig. 5:**
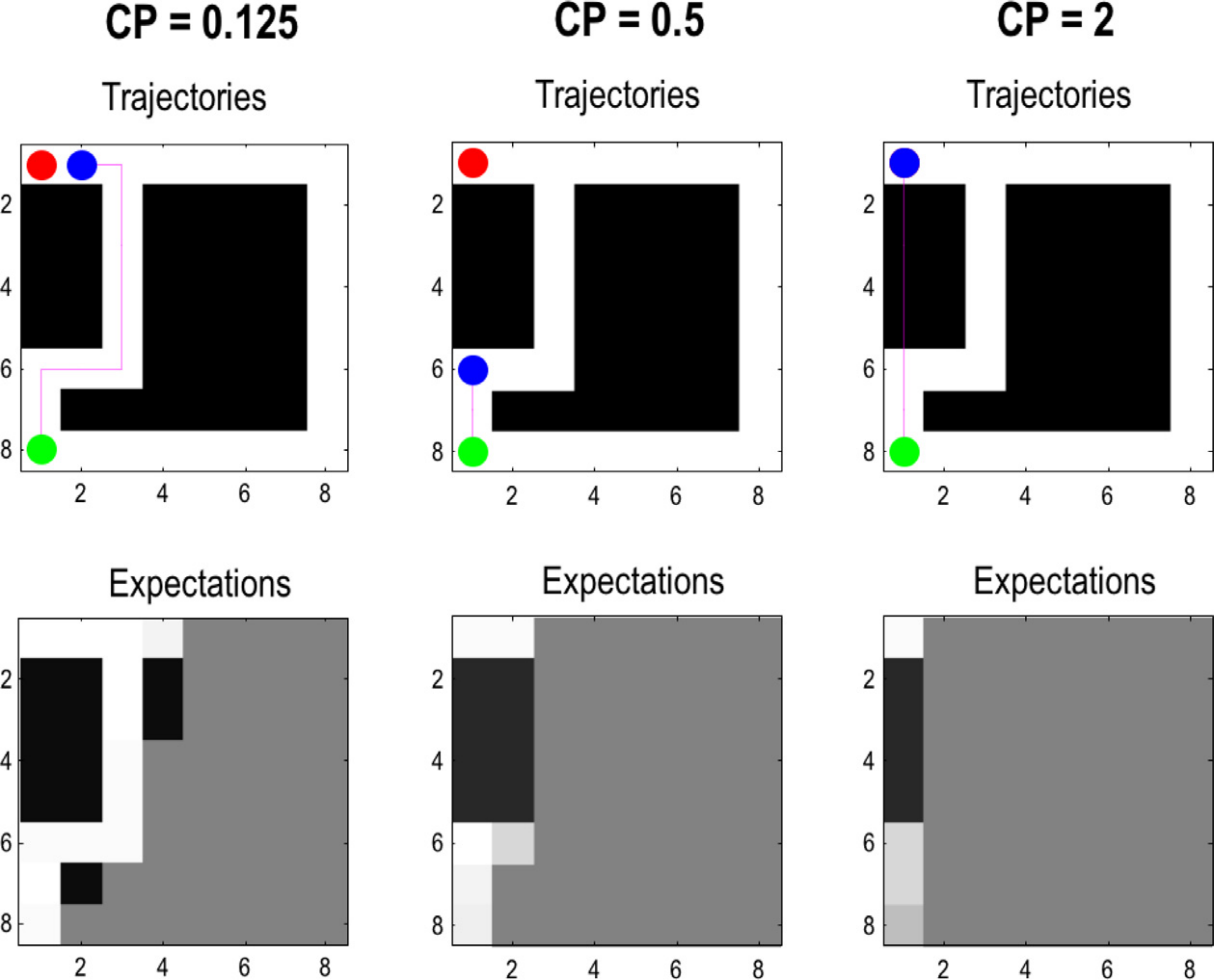
**Dependency on concentration parameters**: The figures show the environment (in terms of the likelihood of outcomes at each location) and trajectories (top) and expectations (bottom) after the 4th trial for agents with prior concentration parameters of 1/8, 1/2, and 2 respectively. The expected likelihood (lower row) reports the agent's expectations about the environment (i.e., the expected probability of an open – white – or closed – black – outcome). We see here that with low priors the agent is more sensitive to the outcomes afforded by interaction with the environment and quickly identifies the shortest path to the target that is allowed by the environment. However, as the agent's prior precision increases, it requires more evidence to update its beliefs; giving the environment a chance to respond to the agent's beliefs and subsequent action. In this case, a ‘desire’ path (i.e. shortcut) is starting to emerge after just four trials (see upper right panel). We focus on this phenomenon in the next figure.

Crucially, different prior settings of the concentration parameters lead to qualitatively different behaviours. In Fig. 5 we illustrate the different behaviours the agent exhibits as a function of its initial concentration parameters. This figure shows the trajectories of agents at their fourth trial. The fast-learning, or naïve, agent with low concentration parameters (left) finds the route to the target, where its learning history is shown in Fig. 4. An agent with intermediate concentration parameters (middle) needs more observations to learn a particular location is open or closed. Once it is confident enough that the intervening region - between its current location and its target location – is closed, it will stay put in an open location. The slow-learning, or stubborn, agent with high concentration parameters (right) is, after four trials, convinced that the locations it has visited are closed. In subsequent trials, it will explore a trajectory parallel to its current one, and once it knows these states are also closed, stays put in the same place as the agent with medium concentration parameters. Although all three agents start with the same set of beliefs about the structure of their environment, they each ascribe different levels of confidence to these beliefs. This means that they learn (change these beliefs) at different rates, resulting in qualitatively different behaviours. We will use this simple but fundamental difference among agents or phenotypes to illustrate the remarkable impact these differences in prior beliefs can have on econiche construction in later simu-lations.

### 3.3 The environment adapting to an agent

So far, we have considered a stationary environment. That is to say, an agent can move around and selectively sample from its environment, but not change it.^9^ Things change profoundly when we allow the agent to change the statistical structure of the environment itself. In the following simulations, we parameterized the generative process with a Dirichlet distribution, just as we did for the generative model. In particular, we now have both an observation matrix *A*, embodying what the agent believes about the mapping between locations *s* and observations *o*, and an generative matrix ***A***, denoting the *actual* mapping between locations ***s*** and observations *o*. The update equations for the observation matrix and generative matrix (bold) reflect the implicit symmetry of agent-environment interactions:

$$\begin{array}{cc} {\hat{A}}_{t}= & Dir\left({\hat{a}}_{t}\right){\hat{a}}_{t}=a_{t}+o_{t}⊗s_{t}\\ {\hat{A}}_{t}= & Dir\left({\hat{a}}_{t}\right){\hat{a}}_{t}=a_{t}+\left[\begin{array}{c} 1\\ 0 \end{array}\right]⊗s_{t} \end{array}$$

The concentration parameters *a* of the observation matrix at time *t* are updated by adding + 1 to the concentration parameter of a particular outcome *o_t_* at a particular location *s_t_*. The concentration parameters ***a*** of the generative matrix at time *t* are updated by adding + 1 to the concentration parameter of the *open* outcome at the location that the agent visited. In other words, the more often an agent visits a particular location, the more likely this location will provide the agent with open observations. The motivation behind these update rules was to show how easily so-called *`desire paths’* can emerge: the more a path through long grass is trodden, the more `walkable’ it becomes.

The relative value of the environmental concentration parameters ***a*** determines the probability of a particular location providing the agent with an open or closed observation. In all initial situations, we set the concentration parameters to either a low value (1/8) or a high value (1024). The absolute value of the concentration parameters can be interpreted in exactly the same way as in the generative model; namely, the propensity to update in light of new evidence. Here, the evidence is provided by action of the agent on the environment and the propensity for environmental updates corresponds to the *inertia* of the environment, or the ability of the environment to ‘remember’ the trajectory of the agent. In short, the environment can impress an agent to a greater or lesser extent, depending upon the agent's prior beliefs. In exactly the same way, and environment may be, literally, impressed by an agent – to a greater or lesser extent. The degree of ‘impression’ in both cases rests upon the prior precisions encoded by (in this example) prior concentration parameters in the generative model (agent) and generative process (environment) respectively.

**Fig. 6:**
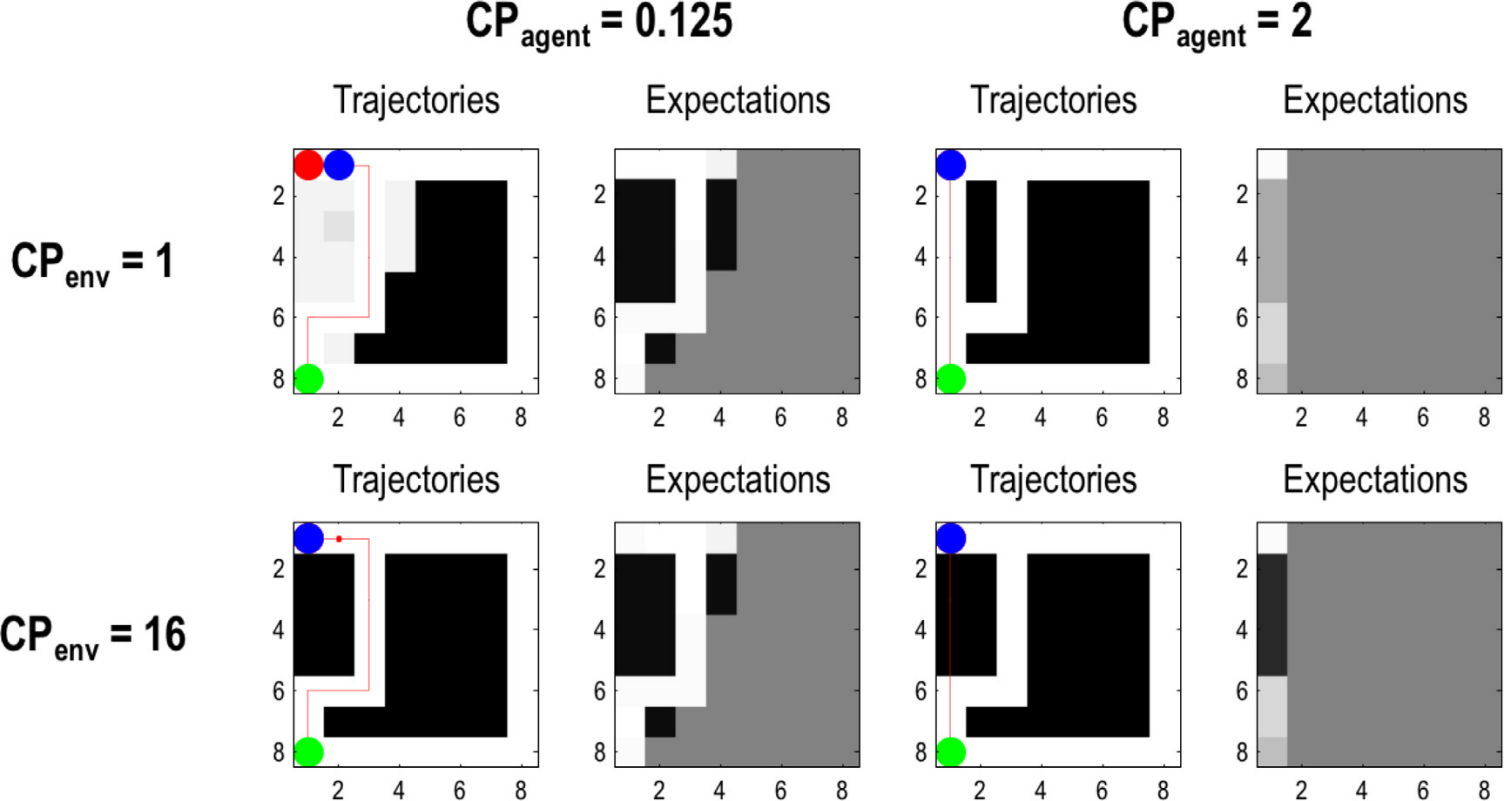
**Dependency on concentration parameters of the agent and environment:** This figure shows the layout of the environment (**A**-matrix) and the agent's expectations about the environment (A-matrix) at the end of the 4th trial, as a function of the prior concentration parameters of both the agent and the environment. The left and right columns show the trajectory for high and low learning rates for the agent (with prior concentration parameters of 1/8 and 2), respectively. The top and bottom row show the trajectory for high and low learning rates of the environment (prior concentration parameters of 1 and 16), respectively. Note the unambiguous emergence of a ‘desire’ path in all scenarios apart from an environment with high concentration parameters and an agent with low concentration parameters (bottom left); i.e., an agent who is willing to learn but an environment that is not yielding. The most unambiguous desire path is clearly evident when the agent is relatively fastidious (with high prior concentration parameters) and the environment is compliant (with low concentration parameters (upper right).

Fig. 6 shows the effects of the different prior concentration parameters on the dynamics of both the agent's observation matrix *A* and the environmental generative matrix ***A***. As above, this Figure shows the path at the fourth trial, as well as the underlying *A* and ***A*** at the end of the fourth trial. The bottom row is similar to Fig. 5: when the environment has high concentration parameters, the agent takes a very long time to change the statistics of the environment. The upper left panels report the situation where concentration parameters are low for both the agent and the environment. The trajectory of the agent over the four trials is identical to the trajectory of the agent with high environmental concentration parameters (bottom right). Since the agent learns a location is closed at once, it never revisits the location to confirm its beliefs, and will therefore not learn about the environmental changes. Although a more efficient path has become available, the agent is unable to exploit this path because the agent places too much confidence in its past experience to explore alternative policies; i.e., its prior beliefs have precluded openness to any epistemic affordance. The upper right panels report the context where concentration parameters are high for the agent, and low for the environment. Like all agents, the agent starts out heading directly for the target state, but in so doing changes the generative matrix ***A*** so that it is more likely to provide the agent with open observations. Because the learning rate of the agent is slower than the rate of change of the environment, the agent carves out an open path by moving repeatedly down the same path (without knowing it has done so).

In summary, depending on the prior concentration parameters of both the agent and the environment, the agent either 1.) learns (and consolidates) the initial path through its environment, 2.) learns the initial path through its environment, but, in learning, opens up new paths, 3.) does not learn an open path or 4.) carves out a new path to its target location.

### 3.4 Agent-environment convergence

Over time, the agent learns the structure of its environment while the environment accumulates knowledge about the agent's behaviour, which depends – in a circular fashion – on the agents expectations. We can quantify the implicit coupling between the agent and environment by exploiting the symmetry between the generative matrix ***A*** and observation matrix *A*. This symmetry allows us to create a `phenotypic space’ that is shared by the agent and environment; namely, the patterns of concentration parameters (of both the generative and observation matrix) that show the greatest changes over time. This phenotypic space can be constructed by generating a covariance matrix consisting of the concatenated generative and observation matrices over time and over trials. The patterns through phenotypic space can be obtained efficiently as the principal components or eigenvectors of the covariance matrix between expectations in both matrices over time.

These eigenvectors define a metric space that summarizes expectations about the consequences of being in any particular location. Crucially, this space is shared by the agent and environment, which allows us to plot the evolution of the agent – and the environment – in the same space and ask how they move in relation to one another. Furthermore, we can visualize the influence of the environment on the agent and vice versa in a compact form via trajectories in this phenotypic space. We will use the (space spanned by the) first two eigenvectors to depict the coupling between the agent and the environment. We can focus our analysis on the first two eigenvectors, because they together capture 98% of the variance. For ease of visualization, we used the deviations from the final expectations of the agent and environment for each simulation. This ensures that their respective trajectories converge on the same point in phenotypic space.

**Fig. 7:**
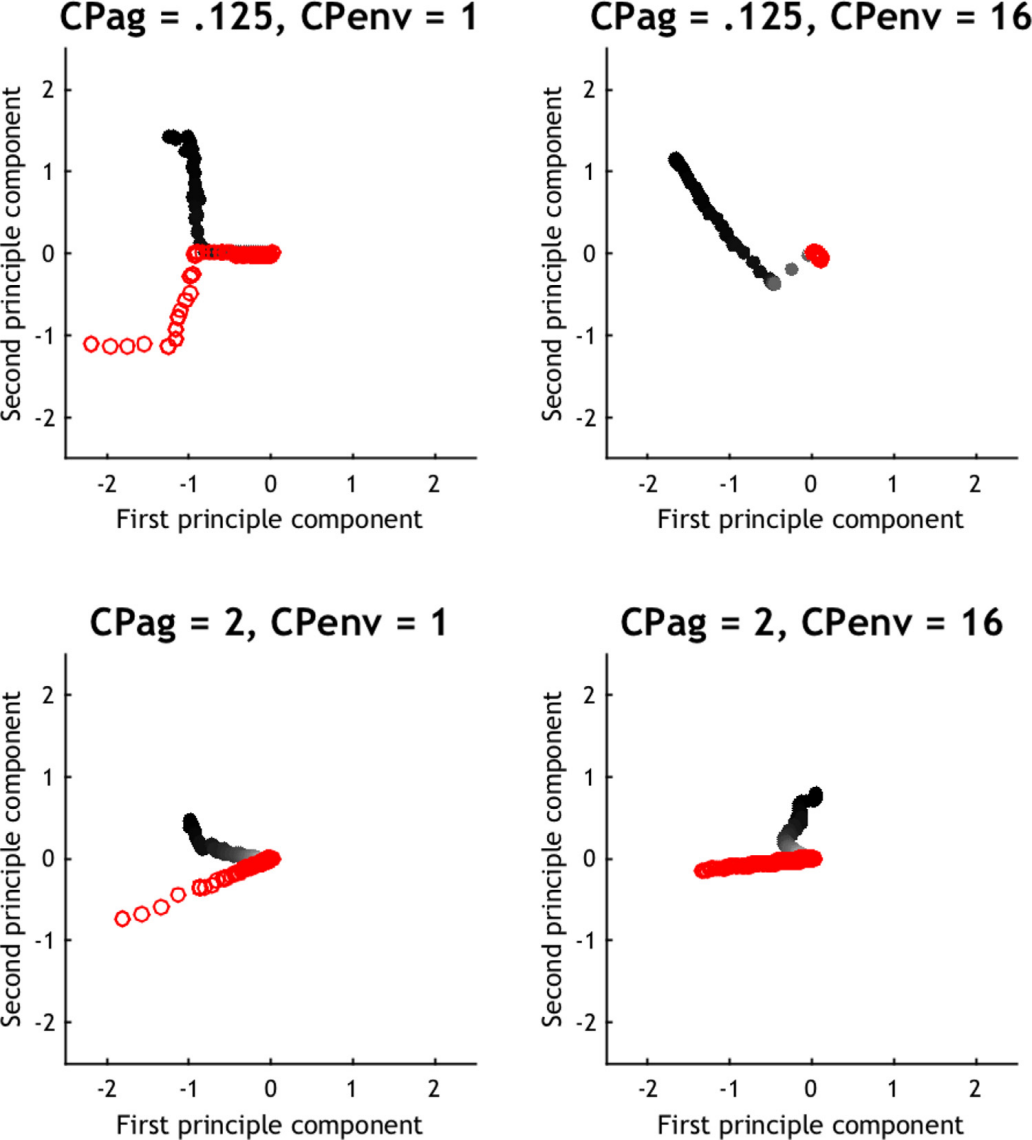
**Trajectories of agent and environment in phenotypic space:** the phenotypic space is defined by the first two eigenvectors of the covariances among the expectations of (both agent and environment) of an open outcome, at each location, over time. The upper and lower panels show the trajectory for low and high prior precision for the agent (with initial concentration parameters of 1/8 and 2), respectively. The left and right panels show the trajectory for low and high prior precision of the environment (with initial concentration parameters of 1 and 16), respectively. Open and closed circles designate the environment and the agent respectively, while the grey scale designates the evolution over time. In this example, the trajectories converge to the same point in phenotypic belief space because the expectations were expressed as deviations from the respective final expectations of the agent and environment.

Fig. 7 plots the corresponding trajectories for the agent (black closed circles) and the environment (red open circles) for each of the four conditions (high and low concentration parameters in the agent and the environment respectively). This licenses a metric interpretation of how the agent's expectations evolve over time (the learning rate), the changes in environmental expectations (the inertia) and the movement of both the agent's expectations and the environment, with respect to each other. The upper and lower panels show the trajectory for low and high prior precision for the agent (with initial concentration parameters of 1/8 and 4), respectively. The left and right panels show the trajectory for low and high prior precision of the environment (with initial concentration parameters of 1 and 16), respectively. Open and closed circles designate the environment and the agent respectively, while the grey scale designates the evolution over time.

The key thing to take from these results is the relative excursion of the environment and agent in their shared phenotypic spaces. It is immediately apparent that the relative prior precision of (implicit) beliefs held by the agent and environment determine how much they move in this space. For example, when both have a low prior precision (in terms of concentration parameters) both move substantially through phenotypic space and crucially, converge on the same direction after a sufficient period of time (see upper left panel). What is remarkable here is that the direction through phenotypic space coincides when the environment and agent are sensitive to each other. Conversely, when the environment is less responsive (i.e., has a higher concentration parameters) it moves relatively little in the phenotypic space – while the agent does all the heavy lifting in terms of adapting to the environment. The lower panels show the equivalent excursions when the agent has greater convictions in its prior beliefs (with high prior concentration parameters). The key thing to observe here is that large distances are traversed in phenotypic space and there is a failure to find a common direc-tion.

This example illustrates an interesting and possibly counterintuitive phenomenon; namely, that the learning ‘about each other’ depends in a sensitive way on the relative confidence placed in prior beliefs (i.e., the Dirichlet parameters in this example). This confidence has a profound effect on the rate at which the agent learns about the environment and vice versa – and the degree to which their respective expectations converge.

### 3.5 Fitness and performance

Using the principal components (i.e. eigenvectors) to define a joint phenotypic space allows one to visualize the development or learning trajectories of the agent and environment. However, this does not mean that the metric distance in phenotypic space reflects the ‘fitness’ of the agent-environment system. In terms of fitness, what matters is the time integral of variational free-energy (free action), given the locations that are actually visited (and outcomes experienced). More formally, from the perspective of the free-energy principle fitness corresponds to model evidence (Campbell, 2016, Frank, 2012):

$$\ln P\left(\tilde{o}\right)\approx -F\left(\tilde{s},\pi ,\theta_{i}\right)$$

In other words, the model evidence is scored by the minimum of free-energy, given a set of observations $\tilde{o}$ and a set of parameters *θ_i_* (most notably the *A* –matrix learned after a series of trials). On this interpretation of free energy, one could assess the ‘fitness’ of a range of agents (parameterized by *θ_i_*) for a given set of environmental data $\tilde{o}$. However, the point of this paper is not to interpret evolution and learning as the optimization of parameters given a fixed environment, but rather *as the convergence of both agent and environment as a function of their reciprocal interaction*. The environmental data $\tilde{o}$ is itself dependent on the statistics of the environment ***θ***_***j***_ (specific to a context *j*) and the policies the agent pursues:

$$F_{i,j}=\min_{\tilde{s},\pi }F\left(\tilde{s},\pi |{\tilde{o}}_{ij},\theta_{i}\right)$$

Here,  ${\tilde{o}}_{ij}$ is the sensory data received by agent *i* in environment *j*.

This allows us to evaluate and compare the fitness of each of the four agents in each of the four environments. For simplicity, we have disabled learning of the agent as well as adaptation of the environment. The result is a 4 × 4 matrix that rates the accumulated free-energy for a trial for an agent *i* in environment *j*.

**Fig. 8:**
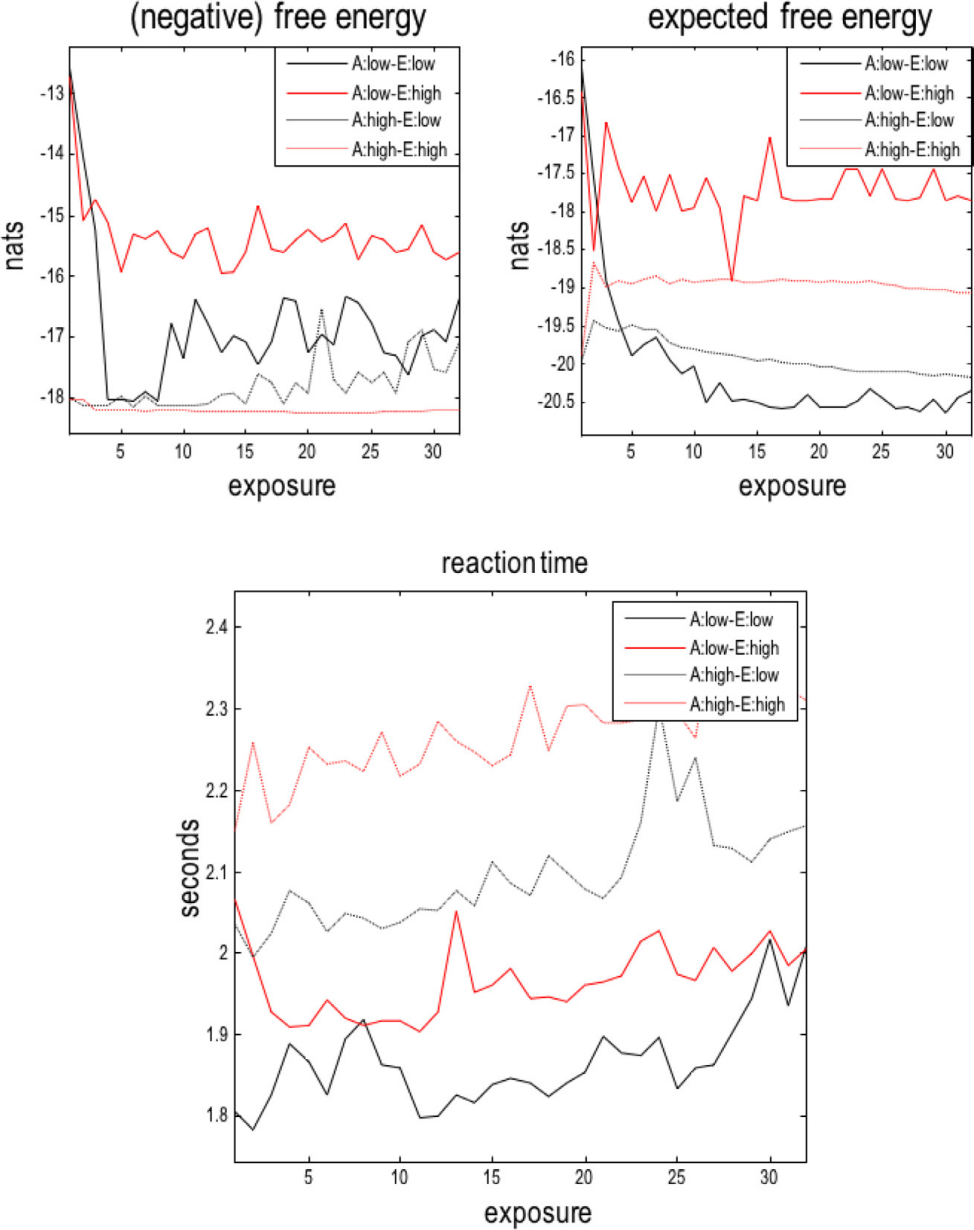
**Temporal evolution of variational and expected free energy:** these graphs report the progressive changes in (negative) variational and expected free energy (upper panels) and simulated reaction times (lower panel) averaged over 16 moves of 32 successive exposures to the environment. The results are shown for an agent with low (solid lines) and high (dotted lines) prior concentration parameters or confidence in its beliefs – in environments with low (black lines) and high (red lines) prior concentration parameters (red lines). (For interpretation of the references to color in this figure legend, the reader is referred to the web version of this article.)

Fig. 8 shows the changes in variational free energy, expected free energy and reaction times (i.e., computational time taken to execute a path) during 32 successive exposures to the environment, where each exposure (or path) comprised 16 moves. These are the same simulations reported in Fig. 7. The solid lines report the changes in free energies and reaction times for agents with low prior concentration parameters or confidence in their beliefs about (location-dependent) outcomes. These can be thought of as relatively naive agents who have little experience of any world. Conversely, the dotted lines refer to agents who are a more experienced (with prior concentration parameters of 4). As above, these two sorts of agents were exposed to environments that were malleable (black lines) and less sensitive to the agent's behaviour (red lines), in virtue of being equipped with prior concentration parameters of 1 and 16 respectively. There are a number of interesting behaviours that these results feature.

First, notice that the free energies fluctuate from exposure to exposure. This reflects the fact that the free energy is a function of sensory encounters that change from moment to moment. The second, perhaps counterintuitive, observation is that the (negative) variational free energy *decreases* on the first few trials. Note that we have plotted negative free energy in the graphs, which can be interpreted as the quality or ‘fitness’ of exchange with the environment. This initial decrease in free energy reflects the fact that the environment is changing and the agents model is playing “catch up”. The key thing here is that the free energy becomes relatively stationary as the agent and environment `get to know each other’. This captures the essence of the variational principle of least of stationary action that underwrites the (nonequilibrium) steady-state that active inference aspires to.

Third, there are some interesting differences between the four simulations. The naive or inexperienced agent in a rigid (high prior concentration parameter) environment appears to fare best – in terms of having the lowest free energy. In other words, the naive agent learns quickly about its unambiguous world and diligently follows the path specified by environmental cues. It therefore avoids all the uncertainty and ambiguity about having to choose between potential shortcuts and the path evidenced by the environment. However, this is not the case for the naive agent in a malleable environment. Here, the environment itself changes as a result of being explored, which means that the agent's generative model is never quite fit for purpose. Although this agent quickly carves out a shortcut, there is a price to be paid in terms of the uncertainty about what will be observed (and what the best course of action is). Note how the black line dips sharply (in the upper left panel) before recovering to steady-state free energy levels.

The more cautious agents (dotted lines) show a different sort of dissociation in terms of free energy. The cautious agent – in a malleable environment – takes a little longer to carve out its shortcut and subsequently learn the consequences of the impressions it leaves on the environment. This results in a slow but progressive decrease in free energy, in contrast to the same sort of agent in a rigid environment – that never quite offers an unambiguous shortcut. As a consequence, the agent is persistently and mildly surprised by the outcomes it encounters. The evolution of expected free energy (shown as negative expected free energy in the figure) follows the same sort of trend. Again, perhaps counterintuitively, the naive agent in a rigid environment appears to be the `happiest’ – in the sense of expecting the lowest free energy, while the naive agent in a compliant environment always expects to be mildly surprised, in virtue of the fact that it keeps changing the environment it is trying to predict.

Finally, the reaction times (i.e., the computational times averaged over all moves that constitute a path) show two interesting features. First, there is a generic increase in computation time with experience. This reflects the fact that the agent's generative model is becoming more precise as it requires experience. The resulting increase in prior precision translates into an increase in complexity and computational cost. This relationship between precision and computational complexity (i.e. reaction time) is mirrored in terms of the differences among the different simulations, with experienced agents expressing the longest reaction times – and environments with greater prior precision appearing to supplement this computational cost. Clearly, these are anecdotal observations; however, they speak to the interesting relationship between the dynamics of perception and the probabilistic fundaments of active inference.

## 4 Conclusion

To summarize, we have presented an active inference scheme that exhibits epistemic foraging, goal-directed behaviour and (unintentional) niche construction using a minimal setup. The key contribution of this paper is to show that *free-energy minimization is a process of the mutual adaptation of agent and environment*: the agent learns from the environment by exploration and the agent's exploration changes the environment until attracting set of states in the agent-environment system is attained. One should note the formal similarity between the update equations for the environment (***A***-matrix) and for the agent (*A*-matrix) used in this paper. Each is parameterized in terms of the underlying concentration parameters of a Dirichlet distribution, and both the agent and the environment ‘accumulate concentration parameters’ at places the agent frequents. Formally speaking, this means that the environment infers or remembers the expectations of the agent in the same way as the agent infers or remembers the layout of the environment. What matters from the perspective of the free-energy principle is the convergence of the agent and environment to a free-energy minimum – that is only defined for a particular agent in a particular environment.

Of course, the agent and environment are not completely symmetric: in the current simulations, the environment is fairly simple and is merely reactive; it does not form expectations about the behaviour of the agent and does not tend to optimize itself by luring the agent into particular behaviours. However, it is not hard to imagine more active niches, for example environments populated with other agents. One can think of an environment consisting of multiple agents, where the sensory states of one agent are generated by the action of the other agents. Over time, the agents mutually constrain each other until an attracting (synchronization) manifold is reached (Friston and Frith, 2015). In such a case, a stubborn agent (one with high concentration parameters) might persist in its behaviour despite evidence to the contrary. In so doing, it forces more flexible agents (with lower concentration parameters– or less confidence in their prior beliefs) to adapt to the behaviour of the confident agent. This makes the behaviour of the confident agent the predominant determinant or ‘driver’ of joint dynamics. This circular causality between an agent and its environment will be an important avenue for future research.

The metaphor of the agent and environment `driving’ each other through phenotypic space, as portrayed in this paper, is in line with extended evolutionary synthesis (Laland et al., 2015). In more traditional approaches to evolutionary biology the fitness landscape is thought of as fixed over time: an agent, or species, is able to scale the peaks to a greater or lesser degree. Extended evolutionary synthesis, on the other hand, is sensitive to the way agents alter their own conditions of existence. On this view, the fitness landscape is not fixed, but co-evolving with the form and affordances of the agent (Walsh, 2014). From an extended evolutionary synthesis perspective, the agent's preferences and conditions of survival also change over phylogenetic and ontogenetic time-scales. In this paper, however, focusing on the emergence of desire paths and niche construction, we have kept the agent's preferences fixed.

Note, finally, that we *could* have equipped the agent with knowledge about how its own actions change the statistics of the environment. This could be done by equipping the agent with beliefs that a change in the *A*-matrix depends on its action. This would lead to a more explicit form of niche construction; behaviour in which agents plan the best route through the environment and then carve out that route. In the present context, this would be less interesting, because everything we want to show (the emergence of adaptive shortcuts or desire paths in the environment), would already be provided to the agent. By not equipping the agent with this knowledge, we can investigate niche construction that emerges from the agent's epistemic foraging and goal-directed behaviour, rather than as the result of planning.

In conclusion, this paper offers a proof-of-principle simulation of niche construction under the free-energy principle. Agent-centered treatments have so far failed to address situations where environments change alongside agents, often due to the action of agents themselves. The key point of this paper is that the minimum of free-energy is not at a point in which the agent is maximally adapted to the statistics of a static environment, but can better be conceptualized an attracting manifold within the joint agent-environment state-space as a whole, which the system tends toward through mutual interaction.

## Declaration

The authors declare that they have no conflicts of interest.

## Appendix A Variational and expected free-energy

We have defined free-energy in terms of a generative model $P(\tilde{o},\tilde{s},\pi ,\theta )\;$and an arbitrary (variational) distribution $Q(\tilde{s},\pi ,\theta )$. The free-energy can be written in several forms to show what its minimization entails:

$$F\left(\tilde{s},\pi ,\vartheta \right)=\underset{divergence}{\underset{︸}{D_{KL}\left[Q\left(\tilde{s},\pi ,\theta \right)∥P\left(\tilde{s},\pi ,\theta |\tilde{o}\right)\right]}}\;-\underset{\log evidence}{\underset{︸}{\ln P(\tilde{o})}}$$

Optimizing the variational distribution $Q(\tilde{s},\pi ,\theta )$ to minimize free-energy implies that the divergence between the variational distribution $Q(\tilde{s},\pi ,\theta )$ and the posterior $P(\tilde{s},\pi ,\theta |\tilde{o})$ is minimized, rendering $Q(\tilde{s},\pi ,\theta )$ an approximate posterior. Furthermore, because the KL-divergence is always greater than zero, minimizing free energy provides an upper bound on the negative log evidence.

$$F\left(\tilde{s},\pi ,\theta \right)=\underset{complexity}{\underset{︸}{D_{KL}\left[Q\left(\tilde{s},\pi ,\theta \right)∥P\left(\tilde{s},\pi ,\theta \right)\right]}}\;-\;\underset{accuracy}{\underset{︸}{E_{Q}\left[\ln P\left(\tilde{o}|\tilde{s},\;\pi ,\theta \right)\right]}}$$

This formulation shows that free-energy is a trade-off between complexity (defined as the divergence between the variational distribution $Q(\tilde{s},\pi ,\theta )$ and the prior $P(\tilde{s},\pi ,\theta )$) and accuracy (defined as surprisal of observations under the variational distribution).

$$F\left(\tilde{s},\pi ,\theta \right)=\underset{energy}{\underset{︸}{-E_{Q\left(\tilde{s},\pi ,\theta \right)}\ln P\left(\tilde{o},\tilde{s},\pi ,\theta \right)}}-\underset{entropy}{\underset{︸}{H\left[Q\left(\tilde{s},\pi ,\theta \right)\right]}}$$

This formulation shows the analogy between variational free-energy and Helmholtz free-energy in thermodynamics. It also shows that the free-energy can be expressed in terms of two quantities that the agent has access to: namely, the (sufficient statistics) of the variational distribution and a generative model.

The generative model is defined as:

$$\begin{array}{ccc} P\left(\tilde{o},\tilde{s},\pi ,\;\theta \right) & = & P\left(\pi |\theta \right)P\left(o_{1}|s_{1},\theta \right)P\left(s_{1}|\theta \right)P\left(\theta \right)\prod_{t=2}^{T}P\left(o_{t}|s_{t},\theta \right)\\ & \times & P\left(s_{t}|s_{t-1},\pi ,\theta \right) \end{array}$$

Where *P*(*θ*) denotes prior probabilities over model parameters, and *P*(*o_t_*|*s_t_, θ*) and $P(s_{t}|s_{t-1},\pi ,\theta )$ denote a likelihood matrix and a probability transition matrix respectively. The outstanding specification of this model question is how the prior over policies *P*(*π*|*θ*) is to be defined.

The logic here is that if an agent expects itself to follow policies that lead to adverse outcomes, the agent would quickly cease to exist. Any agent that does not cease to exist would therefore expect itself to follow policies that it expects to minimize free-energy. This can be expressed by making the prior over policies softmax function of (negative) expected free-energy *G*:

p(π|θ)=σ(−G(π))

The softmax function both introduces a biasing effect based on the effectiveness of the policies and normalizes the expected free-energies: it becomes highly likely that the agent pursues policies that it expects will minimize free-energy into the future.

### Expected free-energy

The expected free-energy for a particular policy is the energy of counterfactual observations and hidden states expected under their posterior predictive distribution *Q*(*o_τ_, s_τ_*|*π*) minus the entropy of the posterior predictive distribution of the hidden states:

$$G\left(\pi \right)=\sum_{\tau }G\left(\pi ,\;\tau \right)$$

$$G\left(\pi ,\;\tau \right)=\underset{energy}{\underset{︸}{-E_{\tilde{Q}}\left[\ln P\left(o_{\tau },s_{\tau }|\pi ,\theta \right)\right]}}-\underset{entropy}{\underset{︸}{H\left[Q\left(s_{\tau }|\pi \right)\right]}}$$

where $\tilde{Q}=Q\left(o_{\tau },s_{\tau }|\pi \right)=P\left(o_{\tau }|s_{\tau }\right)Q\left(s_{\tau }|\pi \right)$. In other words, the expectation $\tilde{Q}$ is over hidden states and outcomes that will be observed in the future (and not over hidden states and policies, as was the case for the variational free-energy). Intuitively, this can be thought of as the free-energy one expects in the future, if one were to pursue a particular policy.

Given $P\left(s_{\tau },o_{\tau },\pi ,\theta \right)=P\left(\;s_{\tau }|o_{\tau },\pi ,\theta \right)P\left(o_{\tau }\right)$ and $\left(\;s_{\tau }|o_{\tau },\pi ,\theta \right)Q\left(o_{\tau }|\pi \right)=P\left(\;o_{\tau }|s_{\tau },\pi ,\theta \right)Q\left(s_{\tau }|\pi \right)$, we can express the expected free energy as (see Appendix A of Friston et al., 2015 for a derivation):

$$G\left(\pi ,\;\tau \right)=\underset{expected\;cost}{\underset{︸}{D_{KL}\left[Q\left(o_{\tau }|\pi \right)P\left(o_{\tau }\right)\right]}}+\underset{expected\;ambiguity}{\underset{︸}{E_{Q}\left[H\left[P\left(o_{\tau }|s_{\tau }\right)\right]\right]}}$$

This expression means that the minimization of *G*(*π*, *τ*) entails minimizing the KL-divergence between (prior) preferred observations and the expected observations under a particular policy (i.e., expected cost) - and minimizing the expected entropy of an outcome under a particular policy (i.e., expected ambiguity). Hence, policies are considered more likely if they realize prior preferences while, at the same time, avoiding ambiguous outcomes that can resolve uncertainty about the hidden or latent states of the world.

## Appendix B Update equations

We have parameterized the generative model as follows:

$$\begin{array}{ccc} P\left(\tilde{o},\tilde{s},\pi ,\;A\right) & = & P\left(\pi \right)P\left(A\right)P\left(s_{1}\right)P\left(o_{1}|s_{1},A\right)\prod_{t=2}^{T}P\left(o_{t}|s_{t},A\right)\\ & \times & P\left(s_{t}|s_{t-1},\pi \right) \end{array}$$

and, using the mean field approximation, we have defined our variational distribution as:

$$Q\left(\tilde{s},\pi ,\;A\right)=Q\left(\pi \right)Q\left(A\right)\prod_{t}^{T}Q(s_{t}\left|\pi \right)$$

We take the following definition of free-energy:

$$F\left(\tilde{s},\pi ,A\right)=\underset{complexity}{\underset{︸}{D_{KL}\left[Q\left(\tilde{s},\pi ,A\right)∥P(\tilde{s},\pi ,A)\right]}}-\underset{accuracy}{\underset{︸}{E_{Q}[\ln P\left(\tilde{o}|\tilde{s},\;\pi ,A\right)}}$$

We can now decompose the free-energy using the conditional independencies in the variational distribution and the generative model:

$$\begin{array}{ccc} F\left(\tilde{s},\pi ,A\right) & = & \sum_{t}E_{Q}\left[F\left(\pi ,t\right)\right]+D_{KL}\left[Q\left(\pi \right)∥P\left(\pi \right)\right]\\ & + & D_{KL}\left[Q\left(A\right)∥P\left(A\right)\right] \end{array}$$

Where:

$$F\left(\pi ,t\right)=\underset{complexity}{\underset{︸}{D_{KL}\left[Q(s_{t}|\pi \right)∥P\left(s_{t}|s_{t-1},\pi \right)]}}-\underset{accuracy}{\underset{︸}{E_{Q}\left[\ln P\left(o_{t}|s_{t}\right)\right]}}$$

Using the facts that P(π)=σ(−G(π)), and $D_{KL}\left[Q\left(x\right)∥P\left(x\right)\right]=E_{Q}\left[\ln Q\left(x\right)-\ln P\left(x\right)\right]$, we can write this as:

$$\begin{array}{ccc} F\left(\tilde{s},\pi ,A\right) & = & E_{Q\left(\pi \right)}\left[F\left(\pi ,t\right)+\ln\pi -G\right]\\ & & +\;E_{Q\left(A\right)}\left[\ln Q\left(A\right)-\ln P\left(A\right)\right]+\ldots \end{array}$$

We can now minimize the free-energy *F* by finding the functional derivates of *F* with respect to all of the elements of the variational distribution $Q(\tilde{s},\pi ,\;\theta )$ and equate them to 0, under the constraint that each of the elements of *Q* expresses a probability distribution (i.e. sums up to one). This is naturally done using Lagrange multipliers (Beal, 2003, Friston et al., 2008). When for example calculating the derivative of *F* with respect to $Q(s_{t^{\prime }}\left|\pi \right)$, we can construct a Lagrangian $\tilde{F}$ using the free-energy expression *F*, a Lagrange multiplier *λ* and the constraint that $\sum_{s}Q(s_{t^{\prime }}\left|\pi \right)$ sums up to 1.

$$\tilde{F}=F-\lambda \left(\sum_{s}Q(s_{t^{\prime }}|\pi )-1\right)$$

We now demand that the functional derivative of the Lagrangian $\tilde{F}$ with respect to $Q(s_{t^{\prime }}\left|\pi \right)$ equals zero, in which case we have found an expression for $Q(s_{t^{\prime }}\left|\pi \right)$ which is both a free-energy minimum and interpretable as a probability distribution.

$$\frac{\partial \tilde{F}}{\partial Q(s_{t^{\prime }}\left|\pi \right)}=0$$

We can now plug in the expression for *F* and do the derivation, in which case we find:

$$-E_{/Q\left(s_{t^{\prime }}|\pi \right)}\ln P\left(\tilde{o},\tilde{s},\pi ,\;\theta \right)+\ln Q(s_{t^{\prime }}|\pi )-1-\lambda =0$$

where $E_{/Q(s_{t^{\prime }}|\pi )}$ designates the expectation with respect to all factors of *Q* except $Q(s_{t^{\prime }}\left|\pi \right)$. Rearranging and combining all terms not dependent on $Q(s_{t^{\prime }}\left|\pi \right)$ in a constant ln *Z*, we find:

$$\begin{array}{ccc} \ln Q(s_{t^{\prime }}\left|\pi \right) & = & \;E_{/Q\left(s_{t^{\prime }}|\pi \right)}[\ln P\left(o_{t^{\prime }}|s_{t^{\prime }}\right)+\ln P\left(s_{t^{\prime }}|s_{t^{\prime }-1},\pi \right)\\ & + & \ln P\left(s_{t^{\prime }+1}|s_{t^{\prime }},\pi \right)]-\ln Z \end{array}$$

Since the only terms that depend and are dependent on $Q(s_{t^{\prime }}\left|\pi \right)$ are the hidden states in the previous and next time step (its Markov blanket), we can write this as:

$$\begin{array}{ccc} \ln Q(s_{t^{\prime }}|\pi ) & = & \;\ln P\left(o_{t^{\prime }}|s_{t^{\prime }}\right)+E_{Q\left(s_{t^{\prime }}-1\right)}\ln P\left(s_{t^{\prime }}|s_{t^{\prime }-1},\pi \right)+E_{Q\left(s_{t^{\prime }}+1\right)}\\ & & \;\;\ln P\left(s_{t^{\prime }+1}|s_{t^{\prime }},\pi \right)-\ln Z \end{array}$$

The transition probabilities *P*(*o_t_*|*s_t_*) and $P(s_{t}|s_{t-1},\pi )$ can be expressed using the *A* and *B* matrices of the generative model (see main text). Filling this in gives:

$$\ln s_{t^{\prime }}^{\pi }=o_{t^{\prime }}\cdot \bar{A}+\bar{B_{t^{\prime }-1}^{\pi }}s_{t^{\prime }-1}^{\pi }-\bar{B_{t^{\prime }}^{\pi }}\cdot s_{t^{\prime }+1}^{\pi }-\ln Z$$

In order to ensure that free-energy is minimized and inference settles on the belief specified by the equation above, we can define the change of current belief ${\dot{s}}_{t^{\prime }}^{\pi }$ as proportional to difference between our current belief $s_{t^{\prime }}^{\pi }$ and the free-energy minimizing belief specified above. The resulting dynamics then perform a gradient descent on free-energy – to settle on the beliefs that minimize free-energy.

$$s_{t^{\prime }}^{\pi }=\sigma \left(v_{t^{\prime }}^{\pi }\right)\;{\dot{v}}_{t^{\prime }}^{\pi }=o_{t^{\prime }}\cdot \bar{A}+\bar{B_{t^{\prime }-1}^{\pi }}s_{t^{\prime }-1}^{\pi }-\bar{B_{t^{\prime }}^{\pi }}\cdot s_{t^{\prime }+1}^{\pi }-v_{t^{\prime }}^{\pi }$$

This is one of the variational update equations denoted in Table 2. The others can be derived in analogous manner. They have a degree of biological plausibility in the sense that they are ordinary differential equations. Note that while the free-energy is minimized separately for each factor of $Q(s_{t^{\prime }}\left|\pi \right)$, the free-energy depends on its Markov blanket $Q(s_{t^{\prime }-1}\left|\pi \right)$, $Q(s_{t^{\prime }+1}\left|\pi \right)$, and observations $o_{t^{\prime }}$, which are themselves minimized. The resulting message passing scheme comprises a series of coupled differential equations that, at each time step t→t+1, is perturbed by an observation $o_{t+1}$. Within that time step, the system relaxes to a new fixed point. By construction, the specifics of the differential equations ${\dot{v}}_{t}^{\pi }$ ensures that the fixed point coincides with the minimum of free-energy. Although it is not the main focus of the current paper, such update equations can be linked to hierarchical message passing in the brain (Friston et al., 2017a).

## Footnotes

1 The Dutch term “olifantenpad” (“elephants’ path”) characterizes the nature of these paths in an imaginative way.

2 As mentioned, reproductive fitness presupposes that the agent is adaptively fit. See Constant et al., (2018) for a more elaborate characterization of the relation between reproductive fitness and adaptive fitness.

3 One might be inclined to associate the agent with a learning rate and the environment with ‘mere’ inertia. Formally, however, we treat the agent and the environment equivalently, both parameterized by concentration parameters.

4 These assumptions are that the system is a weakly-mixing random dynamical system; in other words, a measure preserving system with random fluctuations. The weakly mixing assumption implies a degree of ergodicity; namely, that the system possesses characteristic functions that can be measured.

5 Throughout this paper we will assume discrete time steps and categorical (discrete) states and outcomes.

6 The tilde-symbol (∼) on top of a variable denotes a range of discrete states of that variable over time.

7 ^’^Close’ here is formalised in terms of a Kullback-Leibler divergence between the inferred and true posterior distributions over hidden states in the model. This divergence is the part of the variational free energy that is minimised in active inference. Note that this definition does not actually require the generative model to match the generative process (i.e., econiche) *per se* – just that the observable outcomes it generates can be explained by the generative model.

8 If now what the agent prefers is itself a product of its phylogenetic and ontogenetic history, then what results is akin to an enactive theory of cognition (Allen and Friston, 2016, Bruineberg et al., 2016).

9 In fact, strictly speaking, the simulations did allow the environment to change because we used prior concentration parameters of 4 for the environment. One can see this in the upper panels of Figure 5, which shows the environmental likelihood matrix changes slightly, after four trials or paths.
